# Circulating microRNAs as Potential Biomarkers of Overweight and Obesity in Adults: A Narrative Review

**DOI:** 10.3390/genes16030349

**Published:** 2025-03-17

**Authors:** Francisca Villagrán-Silva, Pía Loren, Cristian Sandoval, Fernando Lanas, Luis A. Salazar

**Affiliations:** 1 Doctoral Program in Morphological Sciences, Faculty of Medicine, Universidad de la Frontera, Temuco 4811230, Chile; f.villagran04@ufromail.cl; 2 Center of Molecular Biology and Pharmacogenetics, Department of Basic Sciences, Faculty of Medicine, Universidad de La Frontera, Temuco 4811230, Chile; pia.loren@ufrontera.cl (P.L.); fernando.lanas@ufrontera.cl (F.L.); 3 Escuela de Tecnología Médica, Facultad de Salud, Universidad Santo Tomás, Los Carreras 753, Osorno 5310431, Chile; cristian.sandoval@ufrontera.cl; 4 Department of Internal Medicine, Faculty of Medicine, Universidad de La Frontera, Temuco 4811230, Chile

**Keywords:** circulating miRNA, genes, overweight/obesity, pathologies, diet, physical habit, adults

## Abstract

In an obesogenic environment, such as the one we have been experiencing in recent decades, epigenetics provides answers to the relationship between hereditary and environmentally acquired patterns that have significantly contributed to the global rise in obesity prevalence. MicroRNA (miRNA) constitutes a diminutive non-coding small RNA molecule, 20 to 24 nucleotides in length, that functions as a regulator of gene regulation at the post-translational level. Circulating miRNAs (c-miRNAs) have been detected in multiple body fluids, including blood, plasma, serum, saliva, milk from breastfeeding mothers, and urine. These molecules hold significant therapeutic value and serve as extracellular biomarkers in metabolic diseases. They aid in the diagnosis and tracking of therapy responses, as well as dietary and physical habit modifications. Researchers have studied c-miRNAs as potential biomarkers for diagnosing and characterizing systemic diseases in people of all ages and backgrounds since then. These conditions encompass dyslipidemia, type 2 diabetes mellitus (T2DM), cardiovascular risk, metabolic syndrome, cardiovascular diseases, and obesity. This review therefore analyzes the usefulness of c-miRNAs as therapeutic markers over the past decades. It also provides an update on c-miRNAs associated with general obesity and overweight, as well as with the most prevalent pathologies in the adult population. It also examines the effect of different nutritional approaches and physical activity regarding the activity of miRNAs in circulation in adults with overweight or general obesity. All of this is done with the aim of evaluating their potential use as biomarkers in various research contexts related to overweight and obesity in adults.

## 1. Introduction

Epigenetics examines heritable alterations in gene activity that arise from phenotypic differences with no DNA sequence changes [1]. Researchers have found many epigenetic mechanisms include post-translational variations of histone proteins, DNA methylation, DNA packaging around nucleosomes, chromatin folding, and microRNAs [2,3,4]. Transgenerational epigenetic inheritance is when these epigenetic changes are passed down from parents to children through somatic cell division [5]. This process explains why gametogenesis and early embryogenesis do not completely erase epigenetic modifications but instead transfer them to the next generation [5,6,7].

In an obesogenic environment, such as the one we have been experiencing in recent decades, epigenetics provides answers to the relationship between hereditary and environmentally acquired patterns that have caused a significant increase in the frequency of overweight/obesity worldwide [8,9]. Regarding the heritable aspects, at the intrauterine level and/or during the lactation stage, the mother’s nutritional lifestyle may influence the programming of fetal development [10]. In terms of those acquired from childhood to adulthood, factors including diet, exercising, environment, and the state or degree of progression of a disease [3,11,12,13] are of great scientific interest. Additional obesity-related characteristics that may modify epigenetic regulation processes include hyperglycemia, uncontrolled endocrine disruptors, hypoxia, oxidative stress, and inflammatory processes, with the resulting secretion of adipokines and cytokines, which could be involved in epigenetic changes that affect insulin sensitivity and adipogenesis [14,15,16,17,18].

It is of interest to our research to specifically study the epigenetics of obesity in relation to the epigenetic mechanisms of microRNAs, particularly circulating microRNAs. Understanding the general aspects of microRNAs, their biogenesis, and the various processes that utilize these biomarkers, particularly in obesity, is crucial. MicroRNAs (or miRNAs) are a class of small non-coding RNA molecules, 20 to 24 nucleotides in length, that function as controllers of gene activity at the post-translational level. In 1993, Lee et al. [19] first identified a small gene called “lin-4”, which does not encode a protein but rather generates a pair of small RNAs responsible for controlling the larval development of the nematode *Caenorhabditis elegans*. Ten years after this discovery, researchers found a link between B-cell leukemia and the number of miRNAs in plants [20,21,22,23], mammals [24,25], and people [26]. Some of the most important things that miRNAs do include controlling post-transcriptional and translational processes that happen in healthy and unhealthy cells. These include studies into cellular death, lipid and fat metabolism, as well as stem cell division and differentiation, and several diseases, including overweight/obesity, cancer, T2DM, osteoporosis and bone metabolism, ischemic heart disease, heart failure, and others [27,28,29,30,31,32,33,34,35].

This review therefore analyzes the usefulness of circulating microRNAs (c-miRNAs) as therapeutic biomarkers over the past decades. It also provides an update on c-miRNAs associated with overweight and general obesity, as well as with the most prevalent pathologies in the adult population. It also examines the influence of different nutritional approaches and exercising on the activity of c-miRNAs in adults with obesity. All of this is done with the aim of evaluating their potential use as biomarkers in various research contexts related to overweight and obesity in adults.

## 2. What Is a Circulating miRNA?

In the human genome, miRNAs represent only 2–3%, which may control the activity of approximately 30–80% of genes [36,37]. Multiple miRNAs could influence the same miRNA, and an individual miRNA may control approximately 200 distinct transcripts, each of which may influence diverse cellular pathways [24,37,38,39]. Humans encode miRNAs in various genomic regions; most introns within protein-coding sequences and non-coding transcripts contain them, although exons also contain them [40,41]. There is a specific way to identify them. In other words, the first three letters of the format hsa-mir-121 stand for the organism, mir-121 serves as the miRNA gene and represents the predicted stem loop region of the primary transcript, and hsa-miR-121 represents the mature miRNA. When the precursor sequences are distinct and the genomic loci that express mature sequences are identical, the names are written as follows: hsa-mir-121-1 and hsa-mir-121-2. A suffix, more specifically a letter, shows mature sequences that are closely related, like hsa-miR-121a and hsa-miR-121b, which both derive from hsa-mir-121a and hsa-mir-121b, which are precursors. Aside from that, if two miRNAs with 22-nt sequences are found to originate from the identical predicted precursors and their relative abundances indicate which miRNA is predominant, they are named miR-56 (the main product) and miR-56* (from the opposite arm of the precursor). However, when the evidence is insufficient to ascertain the predominant sequence, the following designations are given: miR-142-5p (from the 5’ arm) and miR-142-3p (from the 3’ arm). There are exceptions to the format: the miRNAs let-7 and lin-4; these designations are retained for the historic explanations mentioned above [25].

The biosynthesis of miRNAs begins in the nucleus, involving transcription by RNA polymerase II, forming primary miRNAs (pri-miRNAs), long, intramolecular double-stranded RNA in the form of a hairpin with a cap (7mGppp) at 5’ and a poly-adenine (poly-A) tail at 3’. The ribonuclease DROSHA (type III RNA endonuclease) and the RNA-binding protein DGCR8 then integrate the pri-miRNAs into the DROSHA/DGCR8 complex. The cap and poly-A tail of the pri-miRNA are cut by DROSHA into a stem-loop or hairpin fragment of about 60 nucleotides. This makes the precursor miRNA, also called pre-miRNA. XPO5 (Exportin 5) transports pre-miRNAs to the cytoplasm. It is cut by two proteins, DICER endonuclease (also known as type III RNA endonuclease) and TRBP (transactivation response RNA binding protein). This makes a miRNA: miRNA duplex, which is a double-stranded RNA with about 22 nucleotides. The chaperones HSC70 and HSP90 work together to attach the miRNA pair to the AGO protein. Finally, AGO unwinds the miRNA pair, which makes it easier for the guide strand to join the RNA-induced silencing complex (RISC). It then leaders the assembly of miRNA-RISC to the target mRNAs, leading to the degradation of the passenger strand [24,40,42,43,44,45]. Binding happens at a seed sequence of the miRNA, 2–8 from the 5’ end, which combines with the 3’ untranslated regions (3’ UTR) of the target mRNAs. This creates complementary sites that can be controlled by breaking down their target mRNAs and/or stopping their translation [46,47]. These can provide two degrees of complementarity, “perfect or imperfect”, between the miRNA-target base pairing, determining the target transcript’s fate. In the first case, it causes the target to be cut and broken down. In the second case, it does the opposite and silences the mRNA through different means, such as stopping translation, breaking down the mRNA without the cutter, and/or putting it in cytoplasmic processing bodies [45,46,48].

In humans, miRNAs from adipose tissue, including subcutaneous, visceral, and omental (abdominal fat found above the intestine), have as their main function stimulating or inhibiting the differentiation of adipocytes and regulating metabolic homeostasis, inflammatory, lipid metabolism, and endocrine functions, specifically insulin resistance (IR) and appetite regulation, so the deregulation of their expression contributes to the development of obesity and various associated pathologies [18,32,37,49,50]. During miRNA processing, the Dicer enzyme’s abundance plays a crucial role. Research in white adipose tissue (abdominal subcutaneous fat tissue samples) reveals that various aging or premature senescence diseases alter and reduce their levels, leading to a decrease in the levels of multiple miRNAs [51]. According to Ortega et al. [52], donors with obesity and healthy individuals regulated 21 of the 40 differentially expressed miRNAs in differentiated adipocytes in a similar manner. To date, it has not been established exactly how several miRNAs are differentially expressed in white adipose tissue from people living with overweight/obesity; however, fewer than 10 miRNAs are reported as upregulated, while approximately 30 miRNAs are noted as downregulated in this tissue [53]. Establishing a common pattern is challenging due to potential confounding factors like varying tissue preparation protocols, variations in platforms and analysis methods, limited sample sizes, and gender [53,54].

miRNAs are not the only intracellular molecules. Researchers have detected c-miRNAs in various body fluids such as serum, plasma, saliva, urine, and breast milk [55,56,57]. In 2008, research on cancer patients led to the initial description of these molecules in humans [55]. These molecules hold significant therapeutic value and serve as extracellular biomarkers in metabolic diseases. They aid in the diagnosis and tracking of therapy responses, as well as dietary and physical habit modifications [57,58]. c-miRNAs have been studied by researchers as potential biomarkers for diagnosis and characterizing systemic diseases in people of all ages and backgrounds since then. These diseases include T2DM [59], dyslipidemia [60], metabolic syndrome and cardiovascular risk [61], cardiovascular diseases [62], and obesity [63,64,65,66,67].

It is very unlikely that biofluid-based RNases will break down c-miRNAs. This is different from pure RNA, which breaks down quickly [55]. Small vesicles (exosomes, microvesicles, or apoptotic bodies), high-density lipoproteins (HDLs) or low-density lipoproteins (LDLs), or RNA-binding proteins, such as AGO2 (Argonauta 2), can transport miRNAs, explaining this process [68,69].

In the specific case of adipose tissue, the AGO2 protein complex transports miRNA from the same adipose cell to the target mRNA [68,70]. Small exosomes, microparticles, or LDLs move it to the target mRNA in a different organ [68,71,72,73]. Interaction between miRNA and target mRNA influences the translation of key genes and their interaction with other metabolic processes in obesity-linked adipose tissue. These genes control appetite and help adipocytes differentiate in other metabolic processes of adipose tissue [58]. The advantages of using miRNA as biomarkers include detection sensitivity, noninvasive sampling, and multiplexing analysis for increased specificity [74].

### 2.1. Circulating miRNA and General Overweight/Obesity

**Figure 1:**
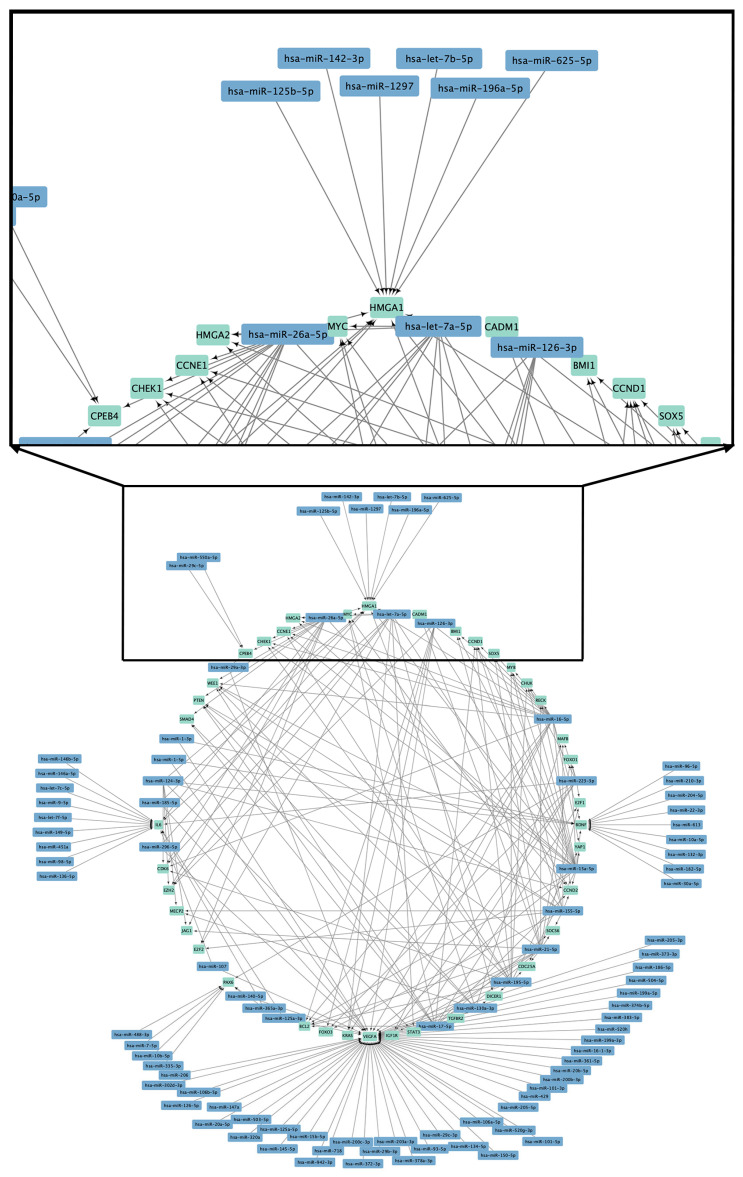
Circulating miRNA in overweight/obesity and potential target. *BCL2:* B-cell lymphoma 2; *BDNF:* Brain-derived neurotrophic factor; *BMI1:* B lymphoma Mo-MLV insertion region 1 homolog; *CADM1:* Cell adhesion molecule 1; *CCND1:* Cyclin D1; *CCND2*: Cyclin D2; *CCNE1:* Cyclin E1; *CDC25A:* Cell division cycle 25A; *CDK6:* Cyclin dependent kinase 6; *CHEK1:* Checkpoint kinase 1; *CHUK:* Conserved helix-loop-helix ubiquitous kinase; *CPEB4:* Cytoplasmic polyadenylation element binding protein 4; *DICER1:* Ribonuclease III; *E2F1:* E2F transcription factor 1; *E2F2:* E2F transcription factor 2; *EZH2:* Enhancer of zeste 2 polycomb repressive complex 2 subunit; *FOXO1:* Forkhead box O1; *FOXO3:* Forkhead box O3; *HMGA1:* High-mobility group AT-hook 1; *HMGA2:* High-mobility group AT-hook 2; *IGF1R:* Insulin-like growth factor 1 receptor; *IL6:* Interleukin 6; *PAX6:* Paired box 6; *JAG1:* Jagged 1; *KRAS:* KRAS proto-oncogene, GTPase; *MAFB:* MAF bZIP transcription factor B; *MECP2:* Methyl-CpG binding protein 2; *MYB:* MYB proto-oncogene, transcription factor; *MYC:* MYC proto-oncogene, bHLH transcription factor; *PAX6:* Paired box 6; *PTEN:* Phosphatase and tensin homolog; *RECK:* Reversion-inducing cysteine rich protein with kazal motifs; *SMAD4:* SMAD family member 4; *SOCS6:* Suppressor of cytokine signaling 6; *SOX5:* SRY-box 5 *STAT3:* Signal transducer and activator of transcription 3; *TGFBR2:* Transforming growth factor beta receptor 2; *VEGFA:* Vascular endothelial growth factor A; *WEE1:* WEE1 G2 checkpoint kinase; *YAP1:* Yes-associated protein 1.

Several investigations have examined modifications in miRNA expression across various metabolic conditions, including IR and overweight/obesity. The studies employed various research models to identify potential biomarkers for predicting T2DM and other cardiovascular diseases. Table 1; Table 2 summarize the miRNAs linked to general obesity in adults and provide a detailed overview of the expression patterns, molecular targets, experimental models, and samples used to establish the association among circulating miRNA and general obesity in adults. The findings highlight the possibility of miRNAs as biomarkers and regulators of obesity, providing insights into the molecular mechanisms associated with obesity-related metabolic dysregulation (Figure 1, Tables S1 and S2).

When comparing healthy and obese subjects, epigenetic studies of c-miRNAs in serum or plasma related with obesity found that obese subjects presented a specific serum miRNA profile. qRT-PCR validation confirmed that the increase in miR-138 and miR-376a (*p* < 0.001) and the decrease in miR-15b are associated with obesity (*p* < 0.001). The ROC curves for miRNAs as miR-15b, miR-138, and miR-376a presented area under the curve (AUC) values of 0.9075 (95% CI: 0.6543–0.9404), 0.9950 (95% CI: 0.9831–1.0000), and 0.8875 (95% CI: 0.7891–0.9860), respectively. Although both miR-15b and miR-376a generated satisfactory ROC values individually, miR-138 showed superior performance as a discriminatory marker between obese patients and healthy control subjects. This suggests that these three miRNAs are potential predictive serum biomarkers of this pathology. Researchers have linked miR-138 and miR-15b to the molecular events of adipogenesis and pancreatic regeneration [63]. During the adipogenic differentiation of multipotent mesenchymal stem cells (MSCs) in humans, miR-138 is downregulated [79]. miR-138 targets the 3’UTR of EID-1, an inhibitor of differentiation that interacts with SHP, an endogenous enhancer of adipogenic *PPARγ2* [80]. Consequently, miR-138 seems to indirectly influence *PPARγ*, a recognized transcription factor that promotes adipogenic gene expression in human MSCs [81]. However, no reports exist regarding the role of miR-376a in obesity. In hepatocellular carcinoma (HCC) cells, miR-376a is significantly downregulated. The elevation of miR-376a represses cell proliferation and induces apoptosis in HCC cells by targeting p85α and directly reducing PIK3R1 [82].

Additionally, in a pilot study conducted in Ireland [64], one of the main objectives was to analyze 10 c-miRNAs from blood samples of 30 individuals living with obesity and 20 non-obese individuals. These miRNAs had been previously evaluated and validated in omental and subcutaneous adipose tissue samples taken from patients living with obesity undergoing bariatric surgery. They discovered a statistically significant decrease in circulating miR-17-5p and miR-132 levels in people living with obesity compared to healthy controls (*p* < 0.024 and *p* < 0.029, respectively). The identified gene targets for miR-132 encompass cAMP response element-binding protein, involved in glucose homeostasis [83], and brain-derived neurotrophic factor, associated with appetite regulation and energy homeostasis [84]. An analysis of the dysregulation of miR-15a-5p and miR-17-5p, followed by target gene prediction [85], revealed significant regulatory interactions with cellular paths, including Wnt, insulin, AMPK, fatty acid metabolism, and TGF-β signaling, proposing a potential role for these miRNAs in the modified transcriptional regulation associated with the advance and progression of complex cardio-metabolic diseases. Research indicates that abnormal expression of miRNAs and their gene targets significantly regulates the development of cardio-metabolic diseases [86]. In fact, a regression model performed by Chen et al. [87] indicates that mir-17-5p is the most significant predictor of metabolic syndrome (MetS) status, exhibiting downregulated activity in subjects with MetS. miR-17-5p functions as an important regulator of the molecular mechanisms that govern insulin secretion and the proliferation and adaptation of pancreatic β-cells under metabolic stress.

In China, research of 40 adult men found a moderate positive association among serum miR-130b levels and body mass index (BMI) (r^2^ = 0.6022, *p* < 0.0001), proposing an association among the quantity of obesity and circulating miR-130b levels. Furthermore, serum miR-130b levels demonstrated the ability to identify people living with overweight or obesity, with a sensitivity and a specificity of 70% and 95%, respectively. ROC curve analysis yielded an AUC of 0.905 (*p* < 0.0001) to discriminate among normal weight individuals and those living with overweight or obesity. These results support the potential of miR-130b as an effective marker of obesity [76]. Regarding other clinical characteristics, serum miR-130b levels showed correlation with body fat percentage assessed by bioimpedance (BF%-BIA; r^2^ = 0.1858, *p* < 0.0035), arm circumference (r^2^ = 0.4597, *p* < 0.0001), triceps skinfold (r^2^ = 0.2043, *p* < 0.0021), calf circumference (r^2^ = 0.3059, *p* < 0.0001), waist circumference (r^2^ = 0.4742, *p* < 0.0001), and triglyceride (TG; r^2^ = 0.1773, *p* < 0.0049) levels. However, no significant correlations were found with diastolic and systolic blood pressure, glucose, total cholesterol (CHOL-C), LDL cholesterol (LDL-C), HDL cholesterol (HDL-C), or insulin. In addition, circulating miR-130b was shown to be a potential biomarker of metabolic disorders, such as metabolic syndrome (AUC 0.833, *p* < 0.001; specificity 96% and sensitivity 55%) and hypertriglyceridemia (AUC 0.758, *p* < 0.01) [76]. TGF-β induces the release of miR-130b from mature adipocytes. Alongside the investigation of miR-130b secretion during adipogenesis, additional signaling factors that may influence the release of miR-130b from mature adipocytes have been analyzed. *PPARγ* ligands, along with cytokines and insulin, are thought to have intricate regulatory effects on adipose tissue in different biological contexts [76].

On the other hand, another study conducted with 120 Chinese adults, aged between 40 and 60 years, identified circulating miR-223 as a biomarker of obesity. Serum miR-223 levels were significantly lower in people living with overweight/obesity compared with people living with normal weight (median: 4.56 vs. 7.54, *p* < 0.001; and 1.06 vs. 7.54, *p* < 0.001, respectively). Furthermore, a logistic regression model adjusted for age, sex, triglycerides (TG), BMI, high-sensitivity C-reactive protein, and CHOL-C showed that people living with obesity, with circulating miR-223 levels less than 1.06 have a 2.12-fold higher risk (95% CI: 1.62–8.4, *p* < 0.05) than those with normal levels [66]. The expression of miR-223 in macrophages plays a crucial role in adipocyte inflammatory and metabolic responses [88]. A prior report indicated that miR-223 activity is downregulated in monocytes and converted in macrophages, leading to increased objective gene activity in the latter and stimulating pro-inflammatory activation [89].

In the United States, a study was conducted whose main objective was to evaluate the effect of overweight/obesity on c-miRNAs related to inflammation (miR-34a, miR-126, miR-146a, miR-150, and miR-181b), independently of other cardiovascular risk factors. The study included 45 sedentary middle-aged adults (47–64 years) of both sexes, distributed according to their BMI in three groups: people living with normal weight (n = 15), people living with overweight (n = 15), and people living with obesity (n = 15). Among the main results, circulating levels of miR-34a were approximately 200% higher in the obese group (2.84 ± 0.92) compared to the normal weight group (1.20 ± 0.29; *p* < 0.05), with no significant differences from the overweight group. Furthermore, a moderate positive association was seen between miR-34a levels and BMI (r = 0.43, *p* < 0.05). Instead, circulating miR-126 levels were approximately 65% lower in the obese (1.02 ± 0.17) and overweight (1.17 ± 0.27) groups compared with the normal weight group (3.41 ± 0.61; *p* < 0.05) and showed a moderate inverse correlation with BMI (r = −0.48, *p* < 0.05). Regarding miR-146a, circulating levels were significantly lower in the overweight (2.19 ± 0.68) and obese (2.71 ± 0.73) groups than in the normal weight group (5.89 ± 1.10; *p* < 0.05) and were mildly inversely correlated with BMI (r = −0.33, *p* < 0.05). miR-150 levels were approximately 60% lower in the obese (0.59 ± 0.16) and overweight (0.59 ± 0.19) groups compared with the normal weight group (1.46 ± 0.32; *p* < 0.05) and were moderately inversely associated with BMI (r = −0.43, *p* < 0.05). It is noteworthy that none of the c-miRNAs analyzed showed an association with the percentage of body fat measured and body mass by dual Energy X-ray Absorptiometry (DXA), systolic and diastolic blood pressure, blood glucose, lipid profile, or insulin levels [75].

The upregulation of miR-34a in endothelial cells correlates with enhanced cytokine production [90], while elevated circulating levels of miR-34a are linked with coronary artery disease [91]. This research confirms and expands upon previous findings, demonstrating a notable (~200%) elevation in circulating miR-34a levels in obese adults relative to those of normal weight. In contrast, circulating miR-126 levels were about 65% lower in overweight and obese adults, which correlated with endothelial inflammation and dysfunction [92,93]. Circulating levels of miR-150 were decreased by approximately 60% in individuals classified as overweight and obese. miR-150 inhibits cytokine production and vascular inflammation by regulating leukocyte and monocyte activation [94]. Alterations in the quantities of miR-34a, miR-126, and miR-150 are related with the heightened inflammatory condition related to obesity [95,96,97].

Adiposity-related inflammation is significantly influenced by the dysregulation of the nuclear factor kappa B (*NF-κB*) pathway, which promotes the production of pro-inflammatory cytokines and vascular adhesion molecules [98]. miR-146a and miR-181b suppress *NF-κB* signaling by downregulating essential proteins necessary for its activation, such as IRAK-1, TRAF-6, and importin-α3 [99,100,101]. Research indicates that elevated levels of circulating miR-146a and miR-181b diminish pro-inflammatory cytokines and adhesion molecules [100]. The expression of miR-146a exhibits an inverse correlation with the levels of *NF-κB*, *TNF-α*, and *IL-6* in adults diagnosed with T2DM [102]. This study identified decreased levels of miR-146a in overweight and obese adults, whereas miR-181b levels did not show significant variation, highlighting the intricate role of miRNA in the inflammatory regulation linked to obesity.

A study conducted in Korea involving 30 overweight/obese and 20 normal-weight adults of both sexes revealed significant differences in the activity of c-miRNAs among both groups. The expression levels of miR-133a (*p* < 0.01), miR-139-5p (*p* < 0.05), miR-15b (*p* < 0.01), miR-26a (*p* < 0.05), miR-301 (*p* < 0.001), miR-30b (*p* < 0.01), miR-30c (*p* < 0.01), miR-374 (*p* < 0.01), miR-451 (*p* < 0.01), miR-570 (*p* < 0.01), and miR-636 (*p* < 0.01) were considerably reduced in the overweight/obese cohort. Conversely, circulating miR-155 exhibited elevated levels in this group (*p* < 0.05). The authors emphasized the notable correlations of circulating miR-15b, miR-26a, and miR-30c with metabolic parameters, supported by prior studies indicating their involvement in obesity and metabolism. miR-15b exhibits a negative correlation with BMI (r = −0.4108, *p* < 0.01), waist circumference (r = −0.289, *p* < 0.05), fasting glucose (r = −0.452, *p* < 0.001), HbA1c (r = −0.352, *p* < 0.05), total fat (r = −0.45, *p* < 0.001), and trunk fat as measured by bioimpedance (r = −0.551, *p* < 0.0001). miR-26a exhibits a negative association with fasting glucose (r = −0.317, *p* < 0.05), HbA1c (r = −0.290, *p* < 0.05), total fat (r = −0.376, *p* < 0.01), and trunk fat (r = −0.44, *p* < 0.01). miR-30c exhibits a negative association with BMI (r = −0.397, *p* < 0.01), fasting glucose (r = −0.473, *p* < 0.001), total fat (r = −0.437, *p* < 0.01), and trunk fat (r = −0.489, *p* < 0.001). The findings highlight the potential of c-miRNAs as biomarkers for evaluating obesity and metabolic disorders [77].

Furthermore, it is essential to report the findings of the other nine c-miRNAs concerning their relationships with metabolic parameters. While miR-133a exhibits an inverse correlation with BMI (r = −0.387, *p* < 0.01), waist circumference (r = −0.514, *p* < 0.0001), fasting glucose (r = −0.519, *p* < 0.0001), HbA1c (r = −0.363, *p* < 0.01), and TG (r = −0.312, *p* < 0.05), it shows a direct relationship exists with LDL-C (r = 0.318, *p* < 0.05). However, an inverse relationship was noted between miR-139-5p and waist circumference (r = −0.326, *p* < 0.05), fasting glucose (r = −0.304, *p* < 0.05), HbA1c (r = −0.363, *p* < 0.01), and C-reactive protein (r = −0.360, *p* < 0.05). Furthermore, a direct correlation exists with HDL-C (r = 0.388, *p* < 0.01). In relation to miR-155, it exhibits an inverse correlation with waist circumference (r = −0.311, *p* < 0.05), HbA1c (r = −0.363, *p* < 0.01), HDL-C (r = −0.334, *p* < 0.05), and total fat (r = −0.291, *p* < 0.05). The analysis indicates a direct correlation with fasting glucose (r = 0.320, *p* < 0.05), HbA1c (r = 0.356, *p* < 0.05), TG (r = 0.475, *p* < 0.001), and C-reactive protein (r = 0.356, *p* < 0.05), while miR-301 exhibits an inverse relationship with BMI (r = −0.50, *p* < 0.01), systolic blood pressure (r = −0.390, *p* < 0.05), total fat (r = −0.351, *p* < 0.05), and trunk fat (r = −0.456, *p* < 0.01). Also, a direct relationship exists with C-reactive protein (r = 0.389, *p* < 0.05). With respect to miR-30b, it has an inverse relationship with BMI (r = −0.412, *p* < 0.01), waist circumference (r = −0.350, *p* < 0.05), fasting glucose (r = −0.457, *p* < 0.001), HbA1c (r = −0.290, *p* < 0.05), total fat (r = −0.339, *p* < 0.05), and trunk fat (r = −0.436, *p* < 0.01). Also, an opposite association was seen between miR-374 and BMI (r = −0.3847, *p* < 0.01), fasting glucose (r = −0.292, *p* < 0.05), HbA1c (r = −0.342, *p* < 0.05), total fat (r = −0.377, *p* < 0.01), and trunk fat (r = −0.445, *p* < 0.01). The analysis found an inverse relationship between miR-451 and BMI (r = −0.390, *p* < 0.01), waist circumference (r = −0.296, *p* < 0.05), fasting glucose (r = −0.360, *p* < 0.05), total fat (r = −0.331, *p* < 0.05), and trunk fat (r = −0.433, *p* < 0.01), while miR-570 has an inverse correlation with total fat (r = −0.404, *p* < 0.01) and trunk fat (r = −0.456, *p* < 0.01); and miR-636 with BMI (r = −0.350, *p* < 0.05), waist circumference (r = −0.344, *p* < 0.05), fasting glucose (r = −0.425, *p* < 0.01), TG (r = −0.352, *p* < 0.05), and C-reactive protein (r = −0.406, *p* < 0.01). The research conducted by Kim et al. [77] underscores the relationship between c-miRNAs and key metabolic parameters.

Recent research has demonstrated a correlation between specific miRNAs and obesity as well as IR. MiR-15b was found to be overexpressed in the liver of diet-induced obese mice, which impaired insulin signaling by targeting the 3′ untranslated region of the insulin receptor [103]. In contrast, the miR-15 family exhibited downregulation in the muscle of diabetic twins, which impacted insulin signaling proteins [104]. MiR-26a, traditionally linked to tumorigenesis, has emerged as a regulator of liver metabolism. It was downregulated in obese mouse models, while its overexpression improved insulin sensitivity and reduced hepatic glucose production [105]. Circulatory miR-26a increased following weight reduction in obese mice [106], and it also promoted thermogenic adipocyte differentiation in humans [107]. Additionally, miR-30c was shown to regulate plasma LDL-C levels, reduce hyperlipidemia in mice, and its levels were decreased in people living with obesity and those with non-alcoholic fatty liver disease [108,109]. Notwithstanding these results, additional studies are required to elucidate the roles of these miRNAs in obesity-related metabolic diseases.

Pathway and ontology analyses indicated that miR-15b, miR-26a, miR-301, miR-30b, and miR-30c participate in essential processes related to obesity, such as adipogenesis, fatty acid oxidation, *mTOR* (mechanistic target of rapamycin kinase) signaling, *PPAR* signaling, and *Wnt* signaling. *PPAR* plays a vital role in adipogenesis [110]. The overactivation of *mTO*R is involved in the development of obesity and IR [111,112]. Wnt signaling also inhibits adipogenesis by maintaining pre-adipocytes [113]. MiR-26a targets various genes in the *mTOR* signaling pathway, while miR-30b, miR-30c, and miR-301 influence Wnt signaling. MiR-15b modulates multiple genes linked to obesity-related processes. The altered expression of these miRNAs in people living with obesity may significantly impact the pathophysiology of obesity, IR, and diabetes.

In a study conducted in Algeria, the relationship of circulating miR-146a and miR-21 with inflammatory conditions was evaluated in 42 adult men divided into two groups: people living with obesity (n = 29) and people living with normal weight (n = 13). The median relative activity of miR-146a and miR-21 was meaningfully lower in people living with obesity compared to people living with normal weight (*p* < 0.001). Regarding inflammatory markers, concentrations of cytokines *IL-6* and *TNF-α* were, on average, higher in the obese group than in the normal group (*p* < 0.001 and *p* < 0.05, respectively). Likewise, in the obese group, a moderate inverse correlation was identified between circulating levels of miR-146a and cytokines *IL-6* (r = −0.44, *p* < 0.05) and *TNF-α* (r = −0.37, *p* < 0.05). Similarly, an opposite relationship was observed among circulating levels of miR-21 and cytokines *IL-6* (r = −0.41, *p* < 0.05) and *TNF-α* (r = −0.50, *p* < 0.01). These findings suggest a possible role of c-miRNAs miR-146a and miR-21 as obesity biomarkers related to the inflammatory state [78]. miR-146a is crucial for the regulation of insulin activity, production, and secretion. Its deregulation in peripheral blood mononuclear cells correlates with diabetes [114,115]. Decreased activity of miR-146a correlates with a proinflammatory condition, resulting in increased quantities of *TNF-α* and *IL-6* in miRNA-deficient mice when exposed to lipopolysaccharide [116]. In human fibroblasts, the overexpression of miR-146a reduces inflammation by inhibiting the secretion of *IL-6* [117]. Additionally, miR-146a and miR-21 suppress *NF-*κB expression and reduce the activity of its target genes, including *IL-6* and *TNF-α*, thereby significantly influencing the modulation of inflammation [118,119].

In contrast, miR-21 acts as an anti-inflammatory mediator and is intricate in the regulation of insulin homeostasis and inflammation [118]. In people living with obesity, a reduction in this factor may facilitate increased insulin secretion by indirectly affecting VAMP2, a key protein in insulin exocytosis [120]. The miR-21 enhances insulin sensitivity in 3T3-L1 cells by promoting the translocation of GLUT4 to the cell membrane [121]. It dynamically regulates inflammation through a negative feedback loop involving *NF-κB* and can be activated by *IL-6* and *STAT3* [122]. In addition, miR-21 has the capacity to diminish aberrant *PPAR-α* signaling; however, its deregulation is implicated in diseases related to obesity, inflammation, and liver disease [123,124].

### 2.2. Circulating miRNAs, Overweight/Obesity, and Associated Pathologies

Overweight/obesity is considered a risk factor for diseases such as dyslipidemia, type 2 diabetes mellitus (T2DM), metabolic syndrome, and cardiovascular diseases. Tables summarize the miRNAs linked to overweight/obesity and associated with metabolic syndrome (Table 3), T2DM (Table 4), dyslipidemia (Table 5), metabolic syndrome and cardiovascular risk (Table 6), cardiovascular disease (Table 7), and insulin resistance and dyslipidemia (Table 8) in adults and provide a detailed overview of the expression patterns, experimental models, and samples used to determine the relationship between c-miRNAs and associated pathologies in adults with overweight/obesity.

A study on T2DM revealed considerable disparities in circulating miR-21 levels across obese and normal weight groups among diabetic and non-diabetic persons [127]. In the diabetic cohort, the relative activity of miR-21 was markedly decreased in people living with obesity compared to that of people living with normal weight (*p* < 0.05). In the non-diabetic cohort, circulating miR-21 levels were 80% diminished in obese people relative to those of normal weight (*p* < 0.01). The relative activity of miR-21 exhibited an inverse connection with waist circumference (r = −0.40, *p* < 0.01) and BMI (r = −0.274, *p* < 0.05) in the diabetes cohort. The findings indicate that obesity may predispose individuals to the future onset of T2DM [127]. Instead, in Mexico, research was realized in adults with T2DM, prediabetes, and normal glycemia. In Mexico, a study was carried out on 133 adults with T2DM, prediabetics, and with normal glycemia, where one of its objectives was to look for the relationship of circulating miR-146a, miR-34a, and miR-375 in the functionality of β cells [128]. Previous studies have documented the involvement of miR-375 in pancreatic growth and cell propagation [131], the relationship among miR-34a and miR-146a in programmed cell death, and the reduction in insulin release due to miR-34a’s inhibition of VAMP2 protein expression [132]. In this research, the relative expressions of circulating miR-146a, miR-34a, and miR-375 were not associated with β-cell function according to HOMA-B in participants with T2DM, but miR-34a was positively linked with HOMA-IR and miR-375 with serum CHOL-C and LDL-C levels. In addition, the degree of obesity in patients with T2DM was analyzed according to their BMI, finding a significant increase in circulating miR-34a in overweight and obese patients compared to normal weight T2DM patients (*p* < 0.05) [128].

In another study, conducted in the USA, a subsample of 150 individuals (51.0 ± 10.0 years, 73.0% women, with a BMI of 33.3 ± 6.6 kg/m^2^ and waist circumference 102.0 ± 14.0 cm) randomly selected from 27 health centers evaluated the longitudinal relationships between circulating miRNAs and weight change at 1 and 2 years in individuals at risk for T2DM who participated in the Diabetes Prevention Program (DPP) trial. They obtained results in the linear mixed model between circulating miRNAs and weight, adjusted for the covariates age, sex, race, and ethnicity; initial weight; and trial arm, a significant association with weight loss over 2 years, miR-15a (β: −0.54; 95% CI: −1.04, −0.02; *p* = 0.041), miR-192 (β: 0.6; 95% CI: 0.15, 1.05; *p* = 0.010), miR-197 (β: 0.67; 95% CI: 0.93, 1.0; *p* = 0.002), miR-23a (β: −0.54; 95% CI: −0.99, −0.07; *p* = 0.022), and miR-320a (β: 0.71; 95% CI: 0.27, 1.16; *p* = 0.002). When correcting for multiple comparisons using the phase discovery rate, only two c-miRNAs (miR-197 and miR-320a) remained significant. And in the model for the 3% probability of weight loss after two years, adjusted for the same variables mentioned above, it was found that the sex of the participants was significantly associated with miR-320c (OR: 2.49; 95% CI: 1.03, 6.14; *p* = 0.044), but when correcting for multiple comparisons, the association was not maintained. In the analysis of targeted mRNAs performed in the study, they found that the c-miRNAs that were associated with weight loss, such as miR-197, have as target genes *IL-8*, *FOXO3*, and mitogen-activated protein kinase 1 (MAPK1), which are related to signaling pathways of inflammation, immunity, and cellular activity; and miR-320a has as target genes *BMI1* and *PTEN* that fulfill functions in the Wnt and *NF-κB* signaling pathways and *PI3K/AKT* signaling process and stress response, respectively [126].

A study of 553 healthy people in Sweden called the Malmö Diet and Cardiovascular Cancer Cohort found that IR, dyslipidemia, and obesity are all linked to higher levels of miR-483-5p in the blood [130]. Miao et al. [60] conducted another study in China, comparing obese adult subjects (aged 18–80 years) of both sexes, with and without dyslipidemia, to detect possible crucial genes and miRNAs associated with dyslipidemia. The amounts of miR-3659 and miR-151a-5p in the blood were significantly higher (*p* < 0.05) in people with dyslipidemia compared to healthy controls. The AUC is also written as the ROC curve or receiver operating characteristic; analyses were used to find out how well these miRNAs could predict dyslipidemia. The most significant results for miR-3659 and miR-151a-5p were 0.806 (95% CI: 0.769-0.844; *p* < 0.001) and 0.769 (95% CI: 0.729–0.808; *p* < 0.001), respectively. This means that miR-3659 was better at diagnosing dyslipidemia than miR-151a-5p (*p* = 0.02). We have concluded that subjects with obesity may have circulating miR-3659 as a possible biomarker of dyslipidemia.

Elmoselhi et al. [129] conducted a study in the field of cardiovascular research to determine whether c-miRNAs could predict early-onset cardiovascular disease (CVD) in individuals from the United Arab Emirates who were overweight and not receiving enough vitamin D (group A1), those who were overweight and had diabetes (group A2), and a healthy-weight, non-diabetic control group (group C1). Previous studies suggested that endothelial dysfunction is the earliest marker of CVD, particularly in risky populations with obesity, vitamin D deficiency, and T2DM [133]. To identify potential miRNAs associated with cardiovascular and metabolic disorders, a bioinformatics study was performed relating to the three groups (A1, A2, and C1). The study identified miR-182-5p, which upregulated its objective gene *CFL1* (Cofilin 1) by 2.5 log_2_-fold change in group A1 compared to C1. This gene is associated with actin filament fragmentation, depolymerization, and host–virus interactions. Also, miR-200c-3p targeted KIAA1432, which was 1.66 log2 times higher in group A1 than in group C1 and is involved in the release and organization of extracellular matrix. MiR-199a-5p targeted *ZNF415* (zinc finger protein 415), which was upregulated by 2.0 log2-fold in group A1 in comparison to C1, and it functions in zinc finger protein 415 and transcription regulation by RNA polymerase II. The target of miR-193a-5p, *MTRNR2L8* (MT-RNR2 like 8), increased 1.76 log_2_-fold change in group A2 compared to C1 and is associated with the execution of programmed cell death and signaling receptor activity. Lastly, miR-155-5p targeted *C9orf78* (chromosome 9 open reading frame 78), which was upregulated by 1.53 log_2_-fold change in group A1 compared to group A2 and is associated with mRNA splicing, processing, and spliceosome activity [129].

A study of miRNAs in the blood exhibited that miR-182-5p and miR-199a-5p quantities were significantly inferior in A1 compared to C1 group (1.524-fold, *p* < 0.0001 for miR-182-5p and 1.992-fold, *p* < 0.0001 for miR-199a-5p, respectively). miR-193a-5p and miR-155-5p levels in the blood were inferior in A2 than in C1 group (70.763-fold, *p* = 0.0002 and 87.674-fold, *p* = 0.0003, respectively). The A1 group showed a reduced expression of miR-200c-3p compared to C1, although this was not significant. Notably, miR-182-5p, miR-193a-5p, and miR-155-5p correlated with brachial and aortic blood pressure, while miR-182-5p, miR-199a-5p, and miR-155-5p showed associations with pulse wave pressure and augmented pressure index [129].

In relation to metabolic syndrome (MetS), in Brazil [61], researchers looked at c-miRNAs and target genes linked to MetS and cardiometabolic risk in obese people. When compared to obese people who did not have MetS (*p* < 0.05), they discovered that 10 miRNAs from the let family (let-7a-1, let-7f-1, let-7g, and let-7i) and other miRNAs (miR-28, miR-30d, miR-155, miR-181a, miR-363, and miR-1839) were downregulated. It is noteworthy that the downregulation of circulating miR-155 is associated with IR, poor glycemic control, and increased cardiometabolic risk related to MetS. Research indicates that c-miRNAs with abnormal expression could serve as indicators for obesity-related metabolic disorders [130].

A recent study by Brandão-Lima et al. [125] investigated plasma miRNA levels in relation to MetS constituents and sex differences in a cohort of 192 Brazilian adults (20–59 years; 87 men and 105 women) recruited from the 2015 Health Survey of São Paulo with Focus on Nutrition (2015 ISA-Nutrition). The authors identified no significant associations between c-miRNAs levels and MetS within the overall sample. Sex-specific analyses indicated that women with MetS exhibited inferior levels of miR-16 and miR-363 compared to their counterparts without the condition (*p* < 0.05).

MiR-16 is implicated in cellular signaling (*AKT3*), metabolic regulation (*PDK4*), mitochondrial function (*SIRT4*), and inflammation (*IKBKB*, which activates *NF-kB*), while miR-363 is linked to glucose metabolism (*MARK1*) and the *NOTCH* signaling pathway. In men, the significant upregulation of circulating miR-let-7c and miR-30a was observed in those with 1–2 and ≥3 MetS risk factors compared to those without risk considerations (*p* < 0.01 and *p* < 0.05, respectively). In women, elevated miR-150 expression was noted in those with 1–2 MetS risk factors compared to those without (*p* < 0.05). Further, IR was associated with increased expression of circulating miR-let-7c (*p* < 0.01), miR-122 (*p* < 0.05), miR-126 (*p* < 0.05), and miR-30a (*p* < 0.05) in the total sample. In women with IR, miR-let-7c (*p* < 0.01) and miR-126 (*p* < 0.05) exhibited significant elevation. No notable differences were detected in c-miRNAs expression among various BMI categories. No important correlations were found between c-miRNA levels and MetS in the overall sample. Sex-specific analyses indicated that women with MetS exhibited inferior levels of miR-16 and miR-363 compared to their counterparts without the condition (*p* < 0.05). The dysregulation of miR-let-7c, miR-30a, and miR-122 is associated with pathways pertinent to energy metabolism, encompassing insulin signaling (*IRS1/2*), adipogenesis (*PPARGC1A*), lipid oxidation (*IGF1*, *PDK4*), stress response (*PRKRA*), and nutrient metabolism (*IGF2BP*). The pathways are linked to obesity, IR, and endothelial dysfunction [125].

### 2.3. Circulating miRNA, Overweight/Obesity, and Physical Activity and Dietary Habits in Adults

miRNAs are diminutive non-coding RNAs, essential in the regulation of gene activity, and are increasingly acknowledged as significant biomarkers and mediators in metabolic and inflammatory processes. Table 9 consolidates findings from in vivo research investigating alterations in c-miRNAs expression following interventions such as aerobic exercise, calorie restriction, low-glycemic index meals, macronutrient-modified diets, and targeted food supplementation.

In dietary and/or sport interventions, a comparative study in 121 subjects [66], where overweight and obese subjects were given a food allowance of 1200–2000 kcal/day according to their reference weight and an outdoor aerobic exercise regimen of at least 30 min for 5 days a week, this intervention lasted 3 months; where miR-223 was specifically measured, because previous studies had shown that it is a potent regulator of cholesterol biosynthesis, uptake, and efflux [142], a significant element of adipocyte metabolic reactions, and a vital controller in the diet-induced adipose tissue inflammatory response and systemic IR [143]. After the intervention, the median expression of circulating miR-223 levels significantly increased in overweight people from 4.56 to 6.54 (*p* = 0.045) and in obese people from 1.06 to 3.23 (*p* = 0.023). Therefore, circulating miR-223 is a good biomarker of obesity and therapeutic response [66].

A study conducted by Heianza et al. [138] with 495 adults of both sexes in the POUNDS Lost trial implemented a 90-min-per-week moderate physical activity intervention alongside varied macronutrient intake. The objective was to evaluate the alteration of circulating miR-128-1-5p in relation to weight loss and its association with adiposity, IR, and energy expenditure in people living with overweight and obesity. The outcomes confirmed that higher baseline quantities of miR-128-1-5p were linked with smaller reductions in body fat percentage, as measured by DXA (β: 0.59 [SE: 0.20] per 1-SD; *p* = 0.004), six months after nutritional interventions. However, no significant correlations were observed between miR-128-1-5p levels and overall adiposity measures, including body weight, waist circumference, or IR. Significant interactions between physical activity and miR-128-1-5p levels were identified, both at baseline and after a six-month intervention. Reductions were observed in HOMA-IR (*p* = 0.012), fasting insulin (*p* = 0.016), body weight (*p* = 0.033), and waist circumference (*p* = 0.004). In terms of dietary interventions, participants following a high-fat, high-protein diet exhibited lower circulating levels of miR-128-1-5p in comparison to those adhering to a low-fat, moderate-protein diet. In the high-fat, high-protein group, elevated levels of miR-128-1-5p were linked to a reduction in resting energy expenditure (β: −41 [SE: 16] per 1-SD increase; *p* = 0.01). The low-fat, moderate-protein group demonstrated simultaneous increases in miR-128-1-5p levels and resting energy expenditure (β: 30 [SE: 15] per 1-SD increase; *p* = 0.044). The findings show that miR-128-1-5p may distress the differentiation and function of white adipose tissue by modulating the activity of adipocyte differentiation indicators, including PPARG, PPARA, and adipokines such as adiponectin (ADIPOQ) and leptin (LEP) [144]. Heianza et al. [138] suggest that circulating miR-128-1-5p is essential in regulating obesity-related metabolic abnormalities and is associated with positive weight loss results after dietary and physical activity interventions.

Manning et al. [134] conducted a study that investigated a very low-calorie intake (<800 kcal/day) over a four-week period with a cohort of 80 obese women in New Zealand. This study evaluated the response of 21 c-miRNAs to acute weight loss, examining differences between obese and normal-weight women (n = 80) and within the obese group before and after the intervention. The findings revealed that only seven c-miRNAs exhibited significant expression changes in the obese group following the very low-calorie diet, compared to the normal-weight group. Specifically, the expression of miR-568 and miR-34a decreased, whereas miR-433-3p, miR-193a, miR-320, miR-30a-5p, and miR-181 showed increased expression. Within the obese cohort, the comparison of c-miRNA expression before and after the acute weight loss intervention indicated the significant downregulation of eight miRNAs (miR-30a-5p, miR-34a, miR-181a, miR-193, miR-208a, miR-320, miR-433-3p, and miR-568) and the upregulation of five miRNAs (miR-126-3p, miR-375, miR-376, miR-499, and miR-642-5p). Furthermore, all the aforementioned miRNAs, except miR-642 in relation to body weight, were significantly associated with anthropometric parameters, including body weight and BMI. The outcomes propose that the c-miRNAs examined may serve as biomarkers for the beneficial effects of weight loss, demonstrating responsiveness to restrictive diets over a four-week duration.

Giardina et al. [135] guided a double-blind, randomized study to examine the effects of three energy-restricted diets—low glycemic index (LGI), high glycemic index (HGI), and low fat—over a six-month period with 103 adults experiencing overweight or obesity (21 men and 82 women). The findings demonstrated that miR-361 was the sole microRNA exhibiting reduced expression in the LGI compared to the HGI diet group. No substantial variations were perceived in the activity of miR-139-3p, miR-411, miR-432, miR-99b, miR-340, miR-423-5p, or let-7c. The authors detected that the decrease in miR-361 activity correlated with a lower body weight, improved IR management, and reduced cardiovascular risk factors. Therefore, the results are likely mediated by the direct interaction of miR-361 with the *SH2B1* (SH2B adaptor protein 1) gene, which is linked to obesity and appears to be affected by the quality of dietary macronutrients [145].

Another comparative dietary intervention study randomized subjects with obesity to a moderately high-protein diet, a low-fat diet, and normal weight for a duration of 16 weeks. A total of 26 miRNAs showing differential expression between individuals with obesity and those of normal weight. Notably, only seven miRNAs showed significant differences between responders and non-responders to the low-fat diet. These included downregulated miRNAs (miR-130a-3p, miR-142-5p, miR-144-5p, miR-15a-5p, miR-221-3p, miR-29c-3p) and one upregulated miRNA (miR-22-3p). In contrast, no significant differences in miRNA expression were observed in the group following the moderately high-protein diet. Furthermore, we additionally obtained significant results in the AUC analyses of an unadjusted model and adjusted for the basal glucose levels, respectively, of seven c-miRNAs, miR-130a-3p [AUC = 0.726; 95% CI (0.555 to 0.896), *p* = 0.025; AUC = 0.774; 95% CI (0.610 to 0.939), *p* = 0.006], miR-142-5p [AUC = 0.712; 95% CI (0.533 to 0.891), *p* = 0.035; AUC = 0.757; 95% CI (0.585 to 0.928), *p* = 0.011], miR-144-5p [AUC = 0.714; 95% CI (0.535 to 0.892), *p* = 0.034; AUC = 0.750; 95% CI (0.581 to 0.919), *p* = 0.013], miR-15a-5p [AUC = 0.678; 95% CI (0.496 to 0.860), *p* = 0.072; AUC = 0.752; 95% CI (0.586 to 0.917), *p* = 0.011], miR-22-3p [AUC = 0.724; 95% CI (0.555 to 0.893), *p* = 0.024; AUC = 0.778; 95% CI (0.619 to 0.936), *p* = 0.005], miR-221-3p [AUC = 0.729; 95% CI (0.558 to 0.900), *p* = 0.023; AUC = 0.760; 95% CI (0.590 to 0.930), *p* = 0.01] and miR-29c-3p [AUC = 0.681; 95% CI (0.495 to 0.866), *p* = 0.073; AUC = 0.760; 95% CI (0.585 to 0.936), *p* = 0.01], which were differentially expressed between responders and non-responders to a low-fat diet, being powerful predictive biomarkers in different responses to a weight loss intervention [67].

In a comparative study of energy-restricted normal intake (NP) and high-protein (HP) animal and vegetable protein diets, crossover (in the same sample for a period of 7 days per diet) was conducted in 16 overweight adult women (35.0 ± 8.7 years) without chronic diseases, with the objective of identifying whether the high protein diet (four portions of lean, fresh beef per day) alters the expression of selected c-miRNAs associated with pathologies such as obesity, T2DM, and cardiovascular disease, compared to an energy-restricted and normal protein diet (one portion of lean fresh beef). No significant differences were evident in the paired comparison of both diets for the expression of the c-miRNAs studied (miR-24-3p, miR-122-5p, miR-126-3p, miR-146a-5p, miR-150-5p, miR-199a-5p, miR-214-3p, miR-223-3p, miR-320a-3p, and miR-423-5p). Regarding cardiometabolic plasma markers, associations with fasting glucose were identified with c-miRNAs, miR-150-5p in the HP diet (r = −0.448, *p* = 0.017), and miR-423-5p in the total sample (r = −0.42, *p* = 0.001) and the HP diet (r = −0.646, *p* = 0.017); C-reactive protein and c-miRNAs, miR-150-5p in total sample (r = 0.682, *p* < 0.000), in the NP diet (r = 0.653, *p* = 0.015) and in the HP diet (r = 0.615, *p* = 0.025), miR-24-3p in total sample (r = 0.607, *p* = 0.001), in the NP diet (r = 0.773, *p* = 0.002) and in the HP diet (r = 0.585, *p* = 0.036), miR-423-5p in total sample (r = 0.614, *p* = 0.001), in the NP diet (r = 0.682, *p* = 0.01) and in the HP diet (r = 0.615, *p* = 0.025), miR-223-3p in total sample (r = 0.599, *p* = 0.04), and HP diet (r = 0.879, *p* = 0.049), and miR-122-5p in total sample (r = 0.784, *p* = 0.003), and HP diet (r = 0.812, *p* = 0.05); IL-6 and c-miRNAs, miR-24-3p in NP diet (r = 0.597, *p* = 0.031), and miR-423-5p in total sample (r = 0.408, *p* = 0.038) and NP diet (r = 0.681, *p* = 0.01), and finally, HOMA2-β was associated with miR-423-5p in the total sample (r = 0.413, *p* = 0.036). The authors suggest that including fresh, lean beef in a healthy, high-protein dietary pattern in the short-term during energy restriction does not negatively influence c-miRNAs associated with the development of cardiometabolic diseases [141].

In the last five years, dietary research has broadened to encompass innovative health intervention strategies. Key examples include the intake of Brazil nuts, which are high in unsaturated fatty acids, vitamins, minerals, and phytochemicals [136]; prolonged fasting [137]; and the use of plant- and animal-based dietary supplements [139,140]. These strategies are designed to reduce the risks linked to chronic conditions such as obesity, T2DM, IR, MetS, and vascular diseases. Reis et al. [136] examined the effects of daily Brazil nut consumption for two months on 25 miRNAs in a cohort of 29 women with obesity and/or MetS, relative to a control group of 25 women. The research demonstrated a notable upregulation of miR-584-5p and miR-454-3p, with levels rising 2.2-fold following consumption. The analysis of genes associated with c-miRNAs indicates that miR-454-3p is connected to the *VDR/RXR* signaling pathway, targeting genes including *RUNX2* (runt-related transcription factor 2), *MXD1* (MAX dimerization protein 1), and *GADD45A* (growth arrest and DNA-damage-inducible alpha protein). MiR-584-5p similarly regulates the expression of NCOR1 (nuclear receptor corepressor 1) and RANTES (C-C motif chemokine ligand 5). The expression activity of miR-454-3p exhibited a positive correlation with plasma selenium concentrations and an inverse relationship with variations in CHOL-C. Subgroup analyses comparing women with and without MetS (n = 23 and n = 31, respectively) revealed significantly lower circulating miR-375 levels in the MetS group (*p* < 0.05). This miRNA is linked to the regulation of insulin secretion pathways. The authors emphasize the potential of miR-584-5p, miR-454-3p, and miR-375 as biomarkers for health and disease, specifically concerning pathways associated with VDR/RXR signaling and the regulation of insulin secretion.

Long-term fasting is utilized as a therapeutic approach for dyslipidemia, hyperglycemia, hypertension, fatty liver disease, and for improving cognition and mood. Ravanidis et al. [137] examined the impact of a controlled long-term fast, which lasted 10 ± 3 days, involving a daily caloric intake of approximately 250 kcal. This intake included 250 ml of freshly squeezed organic juice at midday, 250 mL of vegetable soup in the evening, and 20 g of honey, while participants were allowed ad libitum consumption of 2–3 liters of water or non-caloric infusions. The study focused on the activity of 24 c-miRNAs associated with metabolism and its disorders. Among these, the expression levels of five c-miRNAs (miR-19b-3p, miR-22-3p, miR-142-3p, miR-143-3p, and miR-145-5p) were reduced following fasting, whereas two miRNAs (miR-122-5p and miR-126-3p) exhibited increased expression. The role of each evaluated miRNA was highlighted in the study: miR-19b plays a pro-inflammatory role in obesity, which may be reversed through fasting; miR-22 is predominantly expressed in the liver, conferring metabolic and energetic benefits; miR-142 contributes to systemic reductions in oxidative stress and inflammation; miR-143/145 enhance glucose metabolism; miR-126 is specifically expressed in endothelial cells and is associated with weight loss; and miR-122, which regulates lipid metabolism, exhibits anti-inflammatory and antitumor properties in the liver. Ravanidis et al. [137] concluded that these findings underscore the beneficial effects of fasting on the activity of seven key c-miRNAs with significant roles in systemic metabolism.

Bastos et al. [139], in Brazil, conducted a randomized, double-blind, crossover study where they evaluated whether acute green tea (GT) supplementation and post-consumption of meals high in fat and saturated fat (HFHS) have an effect on biomarkers of inflammatory and oxidative stress and also evaluated the ability of GT to modulate the expression of c-miRNAs in 15 obese adult women (35.5 ± 9.9 years) without comorbidities. The sample was divided into two groups, giving them two placebo capsules or two GTs (738 mg), respectively, at 10:00 pm and with overnight fasting. The next day, in the morning, they collected a blood sample, and they were given an HFHS snack, and after five hours, another blood sample was extracted. After two weeks, they performed the same crossover procedure on the patients. During the protocol, participants were asked not to consume GT derivatives two weeks prior to the study. Among the main results, no significant differences were observed between inflammatory stress biomarkers (total leukocyte count and serum concentrations of *TNF-α*, *IL-6*, and CRP) before and after ingestion of HFHS food, between the placebo and GT groups. Regarding oxidative stress biomarkers, significant differences were observed in the GT group, with decreased catalase activity (*p* < 0.002) and increased glutathione peroxidase activity (*p* < 0.001) after ingestion of HFHS food, compared to the placebo group. Regarding c-miRNAs, they showed that patients who consumed GT had a lower expression of 62 c-miRNAs compared to patients who did not consume GT. The 62 c-miRNAs showed a predictive analysis of 1,757 target genes, of which 5 c-miRNAs (miR-192-5p, miR-373-3p, miR-595, miR-1266-5p, and miR-1297) that regulate genes associated with the *TGF-beta* (transforming growth factor beta), *CARM1* (arginine methyltransferase-associated coactivator 1), *RSK* (p90 serine/threonine ribosomal S6 kinase), and *BMP* (bone morphogenetic proteins) pathways were selected due to a high interaction score (*p* < 0.001). Thus, an excellent analysis strategy is available to increase the probability of finding genes that play an important role in pathways associated with the effects of GT supplementation in obese patients. Bastos et al. [139] state that the results support that c-miRNA levels are modulated by an HFHS meal and a GT supplement, using a highly sensitive, specific, and reproducible probe-based assay.

Hernández-Gómez et al. [140] conducted a study examining the effects of dietary protein source on c-miRNAs expression in patients with obesity and IR. Participants consumed animal-derived protein (calcium caseinate) during one intervention and plant-derived protein (soy protein isolate) during another, with a one-week washout period between interventions. The results demonstrated that 30 min post-consumption of animal protein, there was a notable rise in the levels of c-miRNAs—miR-27a-3p (*p* < 0.01), miR-29b-3p (*p* < 0.01), and miR-122-5p (*p* < 0.001). No significant effect was observed for miR-222-3p or following the consumption of plant protein. In the comparison of miRNA expression at time points of 0, 30, and 60 min for both protein sources, a significantly higher relative expression of miR-27a-3p (*p* < 0.05), miR-29b-3p (*p* < 0.05), and miR-122-5p (*p* < 0.01) was consistently linked to animal protein intake. In contrast, no significant changes were noted for miR-222-3p. Therefore, the c-miRNAs examined are implicated in the regulation of genes essential to insulin signaling pathways, underscoring their potential significance in metabolic regulation.

## 3. Methods

A comprehensive literature review was conducted up to September 2024 using the meta-search engines Pubmed, SciELO, Scopus, Web of Science, and EMBASE. The search utilized the keywords “overweight”, “obesity”, “circulating miRNA”, “physical habits”, “dietary habits”, “BMI”, “body composition”, “anthropometric measurement”, “diabetes”, “hypertension”, “cardiovascular diseases”, and “metabolic syndrome”, combined with the Boolean operators AND and OR. The review included studies performed only on adults. Studies in pregnant women or with diseases such as cancer and atherosclerosis and studies after bariatric surgery (not having undergone any weight loss surgery (bariatric surgery or gastric sleeve)) were excluded.

A search was conducted in the GEO DataSets database (https://www.ncbi.nlm.nih.gov/gds; accessed on 1 September 2024) utilizing the query: (obesity OR overweight AND “circulating miRNA” NOT (cancer OR atherosclerosis)) AND “Homo sapiens” [porgn: __txid9606]. A total of 163 studies were identified in this search, which were then screened, resulting in the exclusion of those that did not meet the inclusion criteria.

A third search strategy employed bioinformatic analysis using the miTarget Link 2.0 web-based software [146]. A total of 52 genes associated with obesity [147] were input into the software to identify miRNAs related to each gene. A total of 133 miRNAs were identified following the filtration for those exhibiting the strongest validation evidence (see Table S1). The miRNAs were screened using meta-search engines to identify c-miRNAs associated with overweight and obesity in adults without other pathologies. Bibliographic evidence identified 23 c-miRNAs associated with overweight and obesity, linked to 12 genes.

To predict target genes and their functions, validated web-based tools TargetScanHuman 8.0 [148,149] and miRDB [150] were employed.

## 4. Scope and Limitations

This review examines miRNAs as biomarkers associated with overweight and obesity in humans. This work presents findings on alterations in c-miRNAs profiles linked to obesity, evaluated by several body composition and BMI assessment methodologies. This study examines the role of miRNAs in seven common diseases, including T2DM, dyslipidemia, and cardiovascular diseases, along with the impacts of seven dietary and exercise treatments. This assessment is limited by variability in research methodologies, demographic attributes of populations, and sample sizes. Thus, the identified characteristics may limit the generalizability of findings and impede the development of standardized miRNA panels for diagnostic or therapeutic applications. Also, the lasting impact of lifestyle changes on c-miRNAs profiles is insufficiently studied, underscoring the need for further long-term research.

## 5. Conclusions

c-miRNAs exhibit considerable potential as biomarkers for comprehending and addressing overweight and obesity in adults, especially for diagnosing obesity-related comorbidities and assessing the efficacy of lifestyle treatments. This review synthesizes evidence from research examining alterations in miRNAs expression associated with general obesity and its related diseases, as well as the impacts of food and physical activity interventions. However, the translation of these findings into clinical practice remains limited by the lack of standardized panels for diagnosis, prognosis, and therapeutic monitoring. Based on the evidence reviewed, we propose clinically useful miRNA panels tailored to specific metabolic conditions.

For example, panels including miR-15b-5p (serum), miR-138 (serum), and miR-376a (serum); or miR-130b (serum), miR-223-3p (plasma), miR-34a (plasma), and miR-126 (plasma) could be useful as diagnostic panels for early detection of obesity-related metabolic dysfunctions, assisting in patient stratification for targeted interventions. While the panels miR-21-5p (serum), miR-34a (serum), and miR-375 (plasma); or miR-146a (serum), miR-483-5p (serum), miR-223 (plasma) could improve early risk assessment for T2DM in people living with obesity and guide preventive strategies.

In relation to the biomarker panels for tracking the efficacy of lifestyle interventions, enabling personalized therapeutic adjustments, miR-223 (plasma), miR-375 (plasma), miR-126-3p (plasma), and miR-130a-3p (plasma); or miR-128-1-5p (plasma), miR-361 (plasma), miR-584-5p (plasma), and miR-454-3p (plasma) could be used. Additionally, the panels miR-155-5p, miR-182-5p, miR-200c-3p, and miR-199a-5p; or miR-3659, and miR-151a-5p could be used for early detection and monitoring of cardiovascular complications in individuals with obesity or metabolic syndrome.

Therefore, despite methodological limitations, the findings demonstrate the therapeutic and diagnostic value of miRNAs in metabolic research. Future research should concentrate on standardizing miRNA detection techniques and examining the long-term impacts of lifestyle changes in order to improve the precision of these biomarkers in clinical and research settings.

## Figures and Tables

**Table 1:** c-miRNAs upregulated during general obesity in adults.

miRNAs	Expression	Target	Model	Country	Sample/Method	References
miR-15b-5p	Up	NR	in vivo: 20 obese adults (17W/3M) and 20 normal weight (10W/10M)	Spain	Serum/RT-qPCR	[63]
miR-34a	Up	NR	in vivo: 45 sedentary middle-aged adults (47–64 years): 15 normal weight (8M/7W); 15 overweight (8M/7W); and 15 obese (7M/8W)	USA	Plasma/RT-PCR	[75]
miR-130b-3p	Up	*CTSK*, *MTMR9*, *INHBB*, *STEAP4*, *TNF*, *IGF1*, *IL6ST*, *SLC2A1*, *ABCA1*, *ESR1*, *EDN1*, *CLOCK*, *PGC-1alfa*, *Cpt1b*	in vivo: 44 adult men overweight/obesity and normal weight	China	Serum/RT-PCR	[76]
miR-155-5p	Up	NR	in vivo: 50 adults (37–63 years): 20 normal weight (5M/25W) and 30 overweight/obese (19M/11W)	Korea	Total blood/RT-PCR	[77]

Expression: It relates to the deregulation of obesity. W: Women; M: Men; *CTSK*: Cathepsin K; *MTMR9*: Myotubularin related protein 9; *INHBB*: Inhibin subunit beta B; *STEAP4*: STEAP4 metalloreductase; *TNF*: Tumor necrosis factor; *IGF1*: insulin like growth factor 1; *IL6ST*: Interleukin 6 signal transducer; *SLC2A1*: Solute carrier family 2 member 1; *ABCA1*: ATP binding cassette subfamily A member 1; *ESR1*: Estrogen receptor 1; *EDN1*: Endothelin 1; *CLOCK*: Clock circadian regulator; *PGC-1alfa*: Progastricsin-1alfa; *Cpt1b*: Carnitine palmitoyltransferase 1B; NR: Not reported.

**Table 2:** c-miRNAs downregulated during general obesity in adults.

miRNAs	Expression	Target	Model	Country	Sample/Method	References
miR-15b-5p	Down	NR	in vivo: 50 adults (37–63 years): 20 normal weight (5M/25W) and 30 overweight/obese (19M/11W)	Korea	Total blood/RT-PCR	[77]
miR-17-5p	Down	NR	in vivo: 30 obese adults and 20 non-obese	Ireland	Total blood/RQ-PCR	[64]
miR-21-5p	Down	*IL-6*, *TNF-alfa*	in vivo: 42 adult men: 13 normal weight (38 ± 1.17 years) and 29 obese (38 ± 0.97 years)	Argelia	Serum/RT-qPCR	[78]
miR-26a	Down	NR	in vivo: 50 adults (37–63 years): 20 normal weight (5M/25W) and 30 overweight/obese (19M/11W)	Korea	Total blood/RT-PCR	[77]
miR-30b	Down	NR	in vivo: 50 adults (37–63 years): 20 normal weight (5M/25W) and 30 overweight/obese (19M/11W)	Korea	Total blood/RT-PCR	[77]
miR-30c	Down	NR	in vivo: 50 adults (37–63 years): 20 normal weight (5M/25W) and 30 overweight/obese (19M/11W)	Korea	Total blood/RT-PCR	[77]
miR-126	Down	NR	in vivo: 45 sedentary middle-aged adults (47–64 years): 15 normal weight (8M/7W); 15 overweight (8M/7W); and 15 obese (7M/8W)	USA	Plasma/RT-PCR	[75]
miR-132	Down	cAMP	in vivo: 30 obese adults and 20 non-obese	Ireland	Total blood/RQ-PCR	[64]
miR-133a	Down	NR	in vivo: 50 adults (37–63 years): 20 normal weight (5M/25W) and 30 overweight/obese (19M/11W)	Korea	Total blood/RT-PCR	[77]
miR-138	Down	*PPARγ2*, *PPARγ*	in vivo: 20 obese adults (17W/3M) and 20 normal weight (10W/10M)	Spain	Serum/RT-qPCR	[63]
miR-139-5p	Down	NR	in vivo: 50 adults (37–63 years): 20 normal weight (5M/25W) and 30 overweight/obese (19M/11W)	Korea	Total blood/RT-PCR	[77]
miR-146a	Down	*NF-κB*, *IRAK1*, *TRAF6*, importin-alfa3	in vivo: 45 sedentary middle-aged adults (47–64 years): 15 normal weight (8M/7W); 15 overweight (8M/7W); and 15 obese (7M/8W)	USA	Plasma/RT-PCR	[75]
miR-146a	Down	*IL-6*, *TNF-alfa*	in vivo: 42 adult men: 13 normal weight (38 ± 1.17 years) and 29 obese (38 ± 0.97 years)	Argelia	Serum/RT-qPCR	[78]
miR-150	Down	NR	in vivo: 45 sedentary middle-aged adults (47–64 years): 15 normal weight (8M/7W); 15 overweight (8M/7W); and 15 obese (7M/8W)	USA	Plasma/RT-PCR	[75]
miR-223-3p	Down	NR	in vivo: 121 adults (40–60 years): 41 normal weight (20M/21W); 40 overweight (19M/21W); and 40 obese (20M/20W)	China	Plasma/RT-PCR	[66]
miR-301	Down	NR	in vivo: 50 adults (37–63 years): 20 normal weight (5M/25W) and 30 overweight/obese (19M/11W)	Korea	Total blood/RT-PCR	[77]
miR-374	Down	NR	in vivo: 50 adults (37–63 years): 20 normal weight (5M/25W) and 30 overweight/obese (19M/11W)	Korea	Total blood/RT-PCR	[77]
miR-376a	Down	NR	in vivo: 20 obese adults (17W/3M) and 20 normal weights (10W/10M)	Spain	Serum/RT-qPCR	[63]
miR-451	Down	NR	in vivo: 50 adults (37–63 years): 20 normal weight (5M/25W) and 30 overweight/obese (19M/11W)	Korea	Total blood/RT-PCR	[77]
miR-570	Down	NR	in vivo: 50 adults (37–63 years): 20 normal weight (5M/25W) and 30 overweight/obese (19M/11W)	Korea	Total blood/RT-PCR	[77]
miR-636	Down	NR	in vivo: 50 adults (37–63 years): 20 normal weight (5M/25W) and 30 overweight/obese (19M/11W)	Korea	Total blood/RT-PCR	[77]

Expression: It relates to the deregulation of obesity. W: Women; M: Men; *IL-6*: Interleukin 6; *TNF-alfa*: Tumor necrosis factor alfa; *PPARγ2*: Peroxisome proliferator-activated receptor γ2; *PPARγ*: Peroxisome proliferator-activated receptor γ; *NF-κB*: nuclear factor kappa B; *IRAK1*: interleukin 1 receptor associated kinase 1; *TRAF6*: TNF receptor associated factor 6; NR: Not reported.

**Table 3:** c-miRNAs associated with metabolic syndrome in individuals living with obesity.

miRNAs	Expression	Model	Country	Sample/Method	Pathology	References
miR-let-7c’	Up °	in vivo: 192 adults (20–59 years): 29 groups 0 MetS (19M/10W), 96 groups 1-2 MetS (38M/58W) and 67 groups ≥ 3 MetS (30M/37W)	Brazil	Plasma/RT-qPCR	Metabolic syndrome	[125]
miR-16’	Down *	in vivo: 192 adults (20–59 years): 29 group 0 MetS (19M/10W), 96 groups 1-2 MetS (38M/58W) and 67 groups ≥ 3 MetS (30M/37W)	Brazil	Plasma/RT-qPCR	Metabolic syndrome	[125]
miR-30a-5p	Up °	in vivo: 192 adults (20–59 years): 29 group 0 MetS (19M/10W), 96 groups 1-2 MetS (38M/58W) and 67 groups ≥ 3 MetS (30M/37W)	Brazil	Plasma/RT-qPCR	Metabolic syndrome	[125]
mir-150	Up *	in vivo: 192 adults (20–59 years): 29 group 0 MetS (19M/10W), 96 groups 1-2 MetS (38M/58W) and 67 groups ≥ 3 MetS (30M/37W)	Brazil	Plasma/RT-qPCR	Metabolic syndrome	[125]
miR-363’	Down *	in vivo: 192 adults (20–59 years): 29 group 0 MetS (19M/10W), 96 groups 1-2 MetS (38M/58W) and 67 groups ≥ 3 MetS (30M/37W)	Brazil	Plasma/RT-qPCR	Metabolic syndrome	[125]

T2DM: Type 2 diabetes mellitus; * Significant differences in relative expression only in women (*p* < 0.05); ° significant differences in relative expression only in men (*p* < 0.05).

**Table 4:** c-miRNAs associated with T2DM in individuals living with obesity.

miRNAs	Expression	Model	Country	Sample/Method	Pathology	References
miR-15a	Down	in vivo: 150 adults (51 ± 10 years): 50 type 2 diabetic (15M/35W), 42 lifestyle (14M/36W) and placebo (12M/38W)	USA	Plasma/RT-qPCR	T2DM	[126]
**miR-21-5p**	Down	in vivo: 87 adults (40-64 years): 45 type 2 diabetic (26M/21W) and 42 non-diabetic subjects (13M/29W)	Iran	Serum/RT-qPCR	T2DM	[127]
miR-23a	Down	in vivo: 150 adults (51 ± 10 years): 50 type 2 diabetic (15M/35W), 42 lifestyle (14M/36W) and placebo (12M/38W)	USA	Plasma/RT-qPCR	T2DM	[126]
**miR-34a**	Up	in vivo: 133 adults (40–64 years): 82 T2DM group (37M/45W), 16 pre-diabetic (10M/6W) and 35 normal-glycemic (24M/11W)	Mexico	Serum/RT-qPCR	T2DM	[128]
miR-192	Up	in vivo: 150 adults (51 ± 10 years): 50 type 2 diabetic (15M/35W), 42 lifestyle (14M/36W) and placebo (12M/38W)	USA	Plasma/RT-qPCR	T2DM	[126]
miR-197	Up	in vivo: 150 adults (51 ± 10 years): 50 type 2 diabetic (15M/35W), 42 lifestyle (14M/36W) and placebo (12M/38W)	USA	Plasma/RT-qPCR	T2DM	[126]
miR-320a	Up	in vivo: 150 adults (51 ± 10 years): 50 type 2 diabetic (15M/35W), 42 lifestyle (14M/36W) and placebo (12M/38W)	USA	Plasma/RT-qPCR	T2DM	[126]
miR-320c	-	in vivo: 150 adults (51 ± 10 years): 50 type 2 diabetic (15M/35W), 42 lifestyle (14M/36W) and placebo (12M/38W)	USA	Plasma/RT-qPCR	T2DM	[126]

T2DM: Type 2 diabetes mellitus.

**Table 5:** c-miRNAs associated with dyslipidemia in individuals living with obesity.

miRNAs	Expression	Model	Country	Sample/Method	Pathology	References
**miR-151a-5p**	Up	in vivo: 857 adults: 433 dyslipidemia (141M/292W) and 424 normal (128M/296W)	China	Plasma/RT-qPCR	Dyslipidemia	[60]
**miR-3659**	Up	in vivo: 857 adults: 433 dyslipidemia (141M/292W) and 424 normal (128M/296W)	China	Plasma/RT-qPCR	Dyslipidemia	[60]

**Table 6:** c-miRNAs associated with metabolic syndrome and cardiovascular risk in individuals living with obesity.

miRNAs	Expression	Model	Country	Sample/Method	Pathology	References
miR-155	Down	in vivo: 80 obese adults: 48 MetS and 32 non-MetS	Brazil	Blood/RT-qPCR	Metabolic syndrome and cardiovascular risk	[61]

**Table 7:** c-miRNAs associated with cardiovascular disease in individuals living with obesity.

miRNAs	Expression	Model	Country	Sample/Method	Pathology	References
**miR-155-5p**	Down	in vivo: 23 adults (40–70 years): 11 control (5M/6W), 9 vitamin D deficiency and obesity group (6M/3W), and 3 vitamin D deficiency, obesity, and T2DM group (3W)	United Arab Emirates	Plasma/RT-qPCR	Cardiovascular diseases	[129]
**miR-182-5p**	Down	in vivo: 23 adults (40–70 years): 11 control (5M/6W), 9 vitamin D deficiency and obesity group (6M/3W), and 3 vitamin D deficiency, obesity, and T2DM group (3W)	United Arab Emirates	Plasma/RT-qPCR	Cardiovascular diseases	[129]
miR-193a-5p	Down	in vivo: 23 adults (40–70 years): 11 control (5M/6W), 9 vitamin D deficiency and obesity group (6M/3W), and 3 vitamin D deficiency, obesity, and T2DM group (3W)	United Arab Emirates	Plasma/RT-qPCR	Cardiovascular diseases	[129]
**miR-199a-5p**	Down	in vivo: 23 adults (40–70 years): 11 control (5M/6W), 9 vitamin D deficiency and obesity group (6M/3W), and 3 vitamin D deficiency, obesity, and T2DM group (3W)	United Arab Emirates	Plasma/RT-qPCR	Cardiovascular diseases	[129]
**miR-200c-3p**	Down	in vivo: 23 adults (40–70 years): 11 control (5M/6W), 9 vitamin D deficiency and obesity group (6M/3W), and 3 vitamin D deficiency, obesity, and T2DM group (3W)	United Arab Emirates	Plasma/RT-qPCR	Cardiovascular diseases	[129]

T2DM: Type 2 diabetes mellitus.

**Table 8:** c-miRNAs associated with insulin resistance and dyslipidemia in individuals living with obesity.

miRNAs	Expression	Model	Country	Sample/Method	Pathology	References
**miR-483-5p**	Up	in vivo: 553 adults of both sexes: 169 incident cardiovascular disease cases, 140 incident diabetes cases and 259 controls	Sweden	Serum/RT-qPCR	IR and dyslipidemia	[130]

IR: Insulin resistance.

**Table 9:** c-miRNAs, obesity and physical and dietary habits in adults.

miRNAs	Expression	Model	Country	Sample/Method	Physical Exercise	Intervention Diet	References
miR-223	Up	in vivo: 121 adults aged 40 to 60 years, comprising 41 individuals of normal weight (20 males/21 females), 40 overweight individuals (19 males/21 females), and 40 obese individuals (20 males/20 females).	China	Plasma/RT-PCR	Engage in aerobic exercise for a minimum of 30 min each day, five days per week. Three-month intervention	A calorie-restricted and low-fat diet of around 1200–2000 kcal/day, contingent upon baseline weight, which is lowered by approximately 300–500 kcal/day. Duration of intervention: 3 months.	[66]
miR-126-3p	Up	in vivo: 160 adult women (20–65 years): 80 normal weight and 80 obese	New Zealand	Plasma/RT-qPCR	-	4-week very-low-calorie diet of <800 kcal/day	[134]
miR-375	Up						
miR-376	Up						
miR-499-5p	Up						
miR-642-5p	Up						
miR-433-3p	Down						
miR-30a-5p	Down						
miR-34a	Down						
miR-181	Down						
miR-193a	Down						
miR-208a-3p	Down						
miR-320	Down						
miR-568	Down						
miR-361	Down	in vivo: 103 adults overweight/obesity (30–60 years): 36 low-glycemic index diet (8M/28W), 36 high-glycemic index (6M/30W) and 31 low-fat diet (7M/24W)	Spain	Plasma/RT-qPCR	-	Low-GI diet: 40% of calories derived from fat, 42% from low-GI carbohydrates, and 18% from protein. High-GI diet: 40% of calories derived from fat, 42% from high-GI carbohydrates, and 18% from protein. Low-fat diet: 30% of calories derived from fat, 52% from high-glycemic index carbohydrates, and 18% from protein. Duration of intervention: 6 months.	[135]
miR-454-3p	Up	in vivo: 54 overweight/obese adult women (18–55 years): 29 Brazil nut group and 25 control group	Brazil	Plasma/RT-qPCR	-	1 Brazil nut (approximately 1261 μg/Se) per day for 2 months.	[136]
miR-584-5p	Up						
miR-375	Down						
miR-22-3p	Up	in vivo: 103 adults (46.6 ± 9.4 years, 36.1% men): 78 individuals with obesity (38 diet 1 and 40 diet 2) and 25 normal weight	Spain	Plasma/RT-qPCR	-	Diet 1: Moderately high-protein regimen: 40% of total calories derived from carbohydrates, 30% from protein, and 30% from fat. Diet 2: Low-fat diet: 60% of total calories derived from carbohydrates, 18% from protein, and 22% from fat. Duration of intervention: 16 weeks. Significant: No initial advised diets contained fewer than 1200 kcal per day.	[67]
miR-15a-5p	Down						
miR-29c-3p	Down						
miR-130a-3p	Down						
miR-142-5p	Down						
miR-144-5p	Down						
miR-221-3p	Down						
miR-122-5p	Up	in vivo: 32 adults (18–70 years): 22M/10W	Spain	Plasma/RT-qPCR	-	Completed a fasting period of 10 ± 3 days. Prior to fasting, respondents consumed a 600-kcal diet consisting of either rice and vegetables or fruits. Caloric consumption: approximately 250 kcal/day (250 ml of freshly squeezed organic juice at noon, 250 ml of vegetable soup in the evening, and 20 g of honey daily). Consume 2–3 liters of water or non-caloric herbal teas per day. The diet consisted of ovo-lacto-vegetarian organic food, ranging from 800 to 1600 kcal per day, during the fasting period.	[137]
miR-126-3p	Up						
miR-19b-3p	Down						
miR-22-3p	Down						
miR-142-3p	Down						
miR-143-3p	Down						
miR-145-5p	Down						
miR-128-1-5p	Up	in vivo: 495 adults in the POUNDS Lost trial (40–60 years): 202M/293W	USA	Plasma/RT-qPCR	PA goal was 90 min/wk of moderate exercise. Habitual PA levels Baecke questionnaire: preintervention and 6 months after the intervention	The macronutrient intake objectives for the four dietary groups were as follows, reflecting a 2-by-2 factorial design: group 1, low fat and average protein, comprising 20% fat, 15% protein, and 65% carbohydrate; group 2, low fat and high protein, consisting of 20% fat, 25% protein, and 55% carbohydrate; group 3, high fat and average protein, including 40% fat, 15% protein, and 45% carbohydrate; and group 4, high fat and high protein, featuring 40% fat, 25% protein, and 35% carbohydrate. Investigators and personnel responsible for measuring results were oblivious to the dietary assignments of the subjects.	[138]
miR-192-5p	Up	In vivo: 15 adult women with obesity (35.5 ± 9.9 years)	Brazil	Plasma/NanoString nCounter	-	Composition of Green Tea Capsule (GT): 738 mg of tea, 615 mg of total catechins (82.75%), 348 mg of epigallocatechin gallate (EGCG) (47.2%), and 13 mg of caffeine (1.77%). High-fat, high-saturated fat (HFHS) meal: 64 g of fat and 30 g of fatty acids, totaling 1067.45 kcal (54% fat, 37% carbohydrates, and 9% protein). The sample was partitioned into two groups, each receiving either two placebo capsules or two GT capsules at 10:00 PM following an overnight fast. The subsequent morning, a blood sample was collected, patients were administered an HFHS snack, and an additional blood sample was extracted five hours later. After two weeks, the patients were transitioned from the procedure. Participants abstained from consuming GT derivatives for two weeks before to the trial.	[139]
miR-373-3p	Up						
miR-595	Up						
miR-1266-5p	Up						
miR-1297	Up						
miR-27a-3p	Up	in vivo: 10 adults with obesity and IR (27.5–43.2 years; 4M/6W)	Mexico	Plasma/RT-qPCR	-	Vegetable protein (soy protein isolate) or animal protein (calcium caseinate) administered in a single dosage of 1 g/kg body weight. All subjects ingested both therapies at separate intervals, with a one-week washout period between them. The shakes were ingested in one dose. Plasma was collected at 0, 30, 60-, 90-, 120-, and 180-min following beverage consumption.	[140]
miR-29b-3p	Up						
miR-122-5p	Up						
miR-222-3p	NS						
miR-24-3p	NS	in vivo: 16 adult women with overweight (35.0 ± 8.7 years)	USA	Plasma/RT-qPCR	-	Isocaloric and energy-restricted diets (−750 kcal) administered for a duration of 7 days each. Standard protein diet: 1240 ± 0 kcal/day, comprising 15.5% protein (1 serving/day or 4 ounces/day of fresh, lean beef), 55.5% carbs, and 31.2% fat. High-protein diet: 1280 ± 10 kcal/day, comprising 38.8% protein (4 servings/day or 15 ounces/day of fresh, lean beef), 39.0% carbs, and 23.2% fat. Protein sources comprise 60.0% of total protein from fresh and lean beef, and 40.0% from plant items including textured soy protein, tofu, and wheat gluten products.	[141]
miR-122-5p	NS						
miR-126-3p	NS						
miR-146a-5p	NS						
miR-150-5p	NS						
miR-199a-5p	NS						
miR-214-3p	NS						
miR-223-3p	NS						
miR-320a-3p	NS						
miR-423-5p	NS						

IR: Insulin resistance; NS: Not significant; PA: Physical activity.

## Data Availability

No new data were created or analyzed in this study. Data sharing is not applicable to this article.

## Supplementary Materials

The following supporting information can be downloaded at: https://www.mdpi.com/article/10.3390/genes16030349/s1, Table S1: Number of studies related to genes associated with overweight/obesity and miRNAs; Table S2: Description of the signaling pathways and functions of potential targets associated with overweight and obesity.
