# Supplementary material for: Circulating microRNAs as Potential Biomarkers of Overweight and Obesity in Adults: A Narrative Review
Source: Genes (Basel). 2025 Mar 17;16(3):349. doi: 10.3390/genes16030349 (PMC11942292; doi:10.3390/genes16030349)
Supplement: Supplementary file 1 [file genes-16-00349-s001.zip › genes-3471423-supplementary.pdf]

genes

MDPI

Table S1. Number of studies related to genes associated with overweight/obesity and miRNAs.

N°	miRNAs	Number reference	Target	Experiments
1	miR-124-3p	13	BDNF<br>HMGA1<br>STAT3	ELISA, LacZ reporter assay, Flow, Immunohistochemistry, Immunoprecipitation, Luciferase reporter assay, Microarray, qRT-PCR, Western blot, immunostaining, Western blotting.
2	miR-16-5p	9	BDNF<br>HMGA1<br>VEGFA	Luciferase reporter assay, qRT-PCR, Western blot, ELISA.
3	miR-26a-5p	9	CPEB4<br>HMGA1<br>IL6	Flow, Immunohistochemistry, Luciferase reporter assay, qRT-PCR, Western blot, Northern blot.
4	miR-15a-5p	7	BDNF<br>HMGA1<br>VEGFA	ELISA, Flow, Chromatin immunoprecipitation, Immunoblot, Immunohistochemistry, Luciferase reporter assay, Microarray, qRT-PCR, Western blot.
5	miR-126-3p	6	VEGFA	Luciferase reporter assay, qRT-PCR, Western blot, Reporter assay.
6	let-7a-5p	5	HMGA1<br>IL6<br>STAT3	Luciferase reporter assay, qRT-PCR, Western blot.
7	miR-130a-3p	5	PPARG<br>PPARGC1A	GFP reporter assay, ChIP-seq, ELISA, EMSA, Northern blot, Immunofluorescence, Luciferase reporter assay, Microarray, qRT-PCR, Western blot.
8	miR-195-5p	5	HMGA1<br>INSR<br>VEGFA	Luciferase reporter assay, Western blot, qRT-PCR, ELISA.
9	miR-1-3p	4	BDNF<br>VEGFA	ELISA, Immunofluorescence, Immunohistochemistry, Luciferase reporter assay, Microarray, qRT-PCR, Western blot.
10	miR-106a-5p	4	STAT3<br>VEGFA	Luciferase reporter assay, qRT-PCR, Western blot, ELISA.
11	miR-106b-5p	4	STAT3<br>VEGFA	Flow, Luciferase reporter assay, qRT-PCR, Western blot, ELISA.
12	miR-130b-3p	4	PPARG<br>PPARGC1A<br>STAT3	Luciferase reporter assay, GFP reporter assay, qRT-PCR, Western blot.
13	miR-140-5p	4	PAX6<br>VEGFA	Luciferase reporter assay, qRT-PCR, Western blot, ELISA.
14	miR-200b-3p	4	ETV5<br>VEGFA	Luciferase reporter assay, qRT-PCR, Western blot.
15	miR-205-5p	4	VEGFA	Flow, Luciferase reporter assay, qRT-PCR, Western blot, Reporter assay.
16	miR-20a-5p	4	PPARG<br>STAT3<br>VEGFA	Luciferase reporter assay, Microarray, Northern blot, qRT-PCR, Western blot, ELISA.
17	miR-20b-5p	4	PPARG<br>STAT3<br>VEGFA	Luciferase reporter assay, qRT-PCR, ELISA, ChIP, Western blot.
18	miR-223-3p	4	IL6<br>PAX6<br>PTBP2<br>STAT3	Immunohistochemistry, Luciferase reporter assay, qRT-PCR, Western blot.

genes

MDPI

19	miR-29a-3p	4	ADAMTS9	Flow, Immunohistochemistry, Luciferase reporter assay, Microarray,
			CPEB4	Northern blot, qRT-PCR, Western blot.
			VEGFA	
20	miR-29b-3p	4	MMP9	Immunohistochemistry, Luciferase reporter assay, qRT-PCR, Western blot.
			STAT3	
			VEGFA	
21	miR-361-5p	4	VEGFA	ELISA, Luciferase reporter assay, qRT-PCR, Western blot.
22	miR-125b-5p	3	HMGA1	Luciferase reporter assay, Western blot.
			STAT3	
23	miR-145-5p	3	VEGFA	Western blot, Immunoblot, Immunohistochemistry, Luciferase reporter
				assay, Northern blot, qRT-PCR.
24	miR-15b-5p	3	INSR	Luciferase reporter assay, qRT-PCR, Western blot, ELISA.
			MMP9	
			VEGFA	
25	miR-17-5p	3	STAT3	Luciferase reporter assay, Microarray, Northern blot, qRT-PCR, Western blot,
			VEGFA	Immunoblot, ELISA.
26	miR-199a-5p	3	VEGFA	Luciferase reporter assay, Microarray, qRT-PCR, Western blot.
27	miR-200c-3p	3	NOS3	Luciferase reporter assay, qRT-PCR, Western blot.
			VEGFA	
28	miR-204-5p	3	BDNF	Immunofluorescence, Luciferase reporter assay, qRT-PCR, Western blot.
			MMP9	
29	miR-21-5p	3	STAT3	Western blot, qRT-PCR.
			VEGFA	
30	miR-27b-3p	3	PPARG	Luciferase reporter assay, qRT-PCR, Western blot.
31	miR-488-3p	3	PAX6	Luciferase reporter assay, qRT-PCR, Western blot, Microarray,
			POMC	Immunoblotting.
			SLC39A8	
32	miR-7-5p	3	PAX6	Luciferase reporter assay, Western blot.
33	miR-491-5p	3	MMP9	Luciferase reporter assay, Microarray, Western blot, Immunohistochemistry,
				qRT-PCR.
34	hsa-let-7c-5p	2	IL6	
			STAT3	Luciferase reporter assay, qRT-PCR, ELISA
35	hsa-miR-1-5p	2	BDNF	
			VEGFA	Luciferase reporter assay, qRT-PCR, Western blot
36	hsa-miR-101-3p	2	VEGFA	Luciferase reporter assay, qRT-PCR
37	hsa-miR-107	2	IL6	Luciferase reporter assay, qRT-PCR, Western blot, Coimmunoprecipitation,
			VEGFA	Chromatin immunoprecipitation, Immunofluorescence microscopy
38	hsa-miR-124-5p	2	STAT3	Luciferase reporter assay, qRT-PCR, Western blot
39	hsa-miR-125a-3p	2	IL6	
			VEGFA	Immunohistochemistry, Luciferase reporter assay, qRT-PCR, Western blot
40	hsa-miR-125a-5p	2	STAT3	ELISA, Luciferase reporter assay, qRT-PCR, Western blot, Immunoblot,
			VEGFA	Immunohistochemistry
41	hsa-miR-132-3p	2	BDNF	
			MMP9	qRT-PCR, Western blotting, Luciferase reporter assay
42	hsa-miR-133b	2	MMP9	
			PTBP2	Luciferase reporter assay, Reporter assay
43	hsa-miR-182-5p	2	BDNF	Luciferase reporter assay, qRT-PCR, Western blotting
44	hsa-miR-185-5p	2	HMGA1	
			VEGFA	Luciferase reporter assay, qRT-PCR, Western blot
45	hsa-miR-21-3p	2	STAT3	
			ZNF608	Luciferase reporter assay, qRT-PCR, Western blot

46	hsa-miR-210-3p	2	BDNF	2DGE, immunoprecipitation, Luciferase reporter assay, Mass spectrometry, Microarray, qRT-PCR, Western blot
47	hsa-miR-23a-3p	2	PPARGC1A	
			STAT3	Immunofluorescence, Luciferase reporter assay, Western blot
48	hsa-miR-24-3p	2	NOS3	Immunocytochemistry, Luciferase reporter assay, qRT-PCR, Western blot
49	hsa-miR-296-5p	2	HMGA1	
			VEGFA	Luciferase reporter assay, qRT-PCR, Western blot
50	hsa-miR-335-3p	2	NOS3	
			PAX6	Luciferase reporter assay, qRT-PCR, Western blot
51	hsa-miR-365a-3p	2	IL6	
			PAX6	Reporter assay, GFP reporter assay, Western blot
52	hsa-miR-451a	2	IL6	Luciferase reporter assay, qRT-PCR, Western blot
53	hsa-miR-485-3p	2	PPARGC1A	Luciferase reporter assay
54	hsa-miR-93-5p	2	VEGFA	ELISA, Luciferase reporter assay, qRT-PCR, Western blot
55	hsa-miR-942-3p	2	MMP9	
			VEGFA	qRT-PCR, Western blot
56	hsa-let-7b-5p	1	HMGA1	Luciferase reporter assay, Microarray, qRT-PCR, Western blot
57	hsa-let-7e-5p	1	MMP9	Luciferase reporter assay, qRT-PCR, Western blot
58	hsa-let-7f-5p	1	IL6	ELISA, Luciferase reporter assay
59	hsa-miR-101-5p	1	VEGFA	Luciferase reporter assay, qRT-PCR, Western blot
60	hsa-miR-10a-5p	1	BDNF	Luciferase reporter assay, qRT-PCR
61	hsa-miR-10b-5p	1	PAX6	Luciferase reporter assay
62	hsa-miR-1181	1	STAT3	Flow, Immunoprecipitation, Luciferase reporter assay, qRT-PCR, Western blot
63	hsa-miR-1234-3p	1	STAT3	Microarray, qRT-PCR, Western blot
64	hsa-miR-126-5p	1	VEGFA	Luciferase reporter assay
65	hsa-miR-1297	1	HMGA1	Flow, Luciferase reporter assay, qRT-PCR, Western blot
66	hsa-miR-133a-5p	1	MMP9	Luciferase reporter assay, qRT-PCR, Western blot
67	hsa-miR-134-5p	1	VEGFA	ELISA, Luciferase reporter assay
68	hsa-miR-135b-5p	1	MTCH2	Luciferase reporter assay, Western blot
69	hsa-miR-136-5p	1	IL6	Immunofluorescence, Luciferase reporter assay, qRT-PCR, Western blot
70	hsa-miR-142-3p	1	HMGA1	Luciferase reporter assay, qRT-PCR, Western blot
71	hsa-miR-143-3p	1	MMP9	qRT-PCR, Western blot
72	hsa-miR-146a-5p	1	IL6	Microarray, Luciferase reporter assay
73	hsa-miR-146b-5p	1	IL6	Luciferase reporter assay, qRT-PCR, Western blot
74	hsa-miR-147a	1	VEGFA	ELISA, Luciferase reporter assay
75	hsa-miR-148a-3p	1	STAT3	Luciferase reporter assay, qRT-PCR, Western blot
76	hsa-miR-149-5p	1	IL6	ELISA, Luciferase reporter assay, qRT-PCR, Western blot
77	hsa-miR-150-5p	1	VEGFA	ELISA, Luciferase reporter assay
78	hsa-miR-155-3p	1	IRAK3	Luciferase reporter assay, Microarray, qRT-PCR, Western blot
79	hsa-miR-155-5p	1	NOS3	Luciferase reporter assay, qRT-PCR, Western blot
80	hsa-miR-16-1-3p	1	VEGFA	qRT-PCR, Western blot
81	hsa-miR-181a-5p	1	STAT3	qRT-PCR, Western blot
82	hsa-miR-186-5p	1	VEGFA	Chromatin immunoprecipitation, Immunoblot, Immunohistochemistry, Luciferase reporter assay, Microarray, qRT-PCR
83	hsa-miR-196a-5p	1	HMGA1	Luciferase reporter assay, qRT-PCR, Western blot
84	hsa-miR-199a-3p	1	VEGFA	ELISA, Luciferase reporter assay, qRT-PCR, Western blot
85	hsa-miR-203a-3p	1	VEGFA	Immunohistochemistry, Luciferase reporter assay, qRT-PCR, Western blot
86	hsa-miR-205-3p	1	VEGFA	ELISA, Luciferase reporter assay, qRT-PCR, Western blot
87	hsa-miR-206	1	VEGFA	ELISA, Immunofluorescence, Immunohistochemistry, Luciferase reporter assay, Microarray, qRT-PCR, Western blot

88	hsa-miR-212-5p	1	MAF	ELISA, Flow, Immunohistochemistry, Luciferase reporter assay, qRT-PCR, Western blot
89	hsa-miR-22-3p	1	BDNF	Luciferase reporter assay, Microarray
90	hsa-miR-27a-3p	1	PPARG	Luciferase reporter assay, qRT-PCR, Western blot
91	hsa-miR-29b-1-5p	1	STAT3	Immunofluorescence, Luciferase reporter assay, qRT-PCR, Western blot
92	hsa-miR-29c-3p	1	VEGFA	Flow, Immunohistochemistry, Luciferase reporter assay, qRT-PCR, Western blot
93	hsa-miR-29c-5p	1	CPEB4	Luciferase reporter assay, Western blot
94	hsa-miR-302a-5p	1	MMP9	ELISA, Western blot
95	hsa-miR-302d-3p	1	VEGFA	ELISA, Luciferase reporter assay
96	hsa-miR-30a-5p	1	BDNF	Luciferase reporter assay
97	hsa-miR-320a	1	VEGFA	Western blot
98	hsa-miR-337-3p	1	STAT3	Luciferase reporter assay, qRT-PCR, Western blot
99	hsa-miR-338-3p	1	MMP9	Western blot, qRT-PCR
100	hsa-miR-340-5p	1	STAT3	Luciferase reporter assay, qRT-PCR, Western blot
101	hsa-miR-34a-5p	1	PRKD1	Luciferase reporter assay, qRT-PCR, Western blot
102	hsa-miR-361-3p	1	SH2B1	Luciferase reporter assay
103	hsa-miR-372-3p	1	VEGFA	ELISA, Luciferase reporter assay
104	hsa-miR-373-3p	1	VEGFA	ELISA, Luciferase reporter assay
105	hsa-miR-374b-5p	1	VEGFA	LacZ reporter assay
106	hsa-miR-375	1	STAT3	Western blot
107	hsa-miR-378a-3p	1	VEGFA	Luciferase reporter assay, qRT-PCR, Western blot
108	hsa-miR-383-5p	1	VEGFA	ELISA, Luciferase reporter assay
109	hsa-miR-410-5p	1	STAT3	ELISA, Luciferase reporter assay, qRT-PCR, Western blot
110	hsa-miR-429	1	VEGFA	Luciferase reporter assay
111	hsa-miR-4516	1	STAT3	Luciferase reporter assay, Microarray, qRT-PCR, Western blot
112	hsa-miR-486-3p	1	MAF	Luciferase reporter assay, qRT-PCR, Western blot
113	hsa-miR-491-5p	1	MMP9	Luciferase reporter assay
114	hsa-miR-503-5p	1	VEGFA	Luciferase reporter assay
115	hsa-miR-504-5p	1	VEGFA	ELISA, Luciferase reporter assay
116	hsa-miR-519a-3p	1	STAT3	Luciferase reporter assay, qRT-PCR, Western blot
117	hsa-miR-519d-3p	1	STAT3	Luciferase reporter assay, qRT-PCR, Western blot
118	hsa-miR-520c-3p	1	STAT3	Luciferase reporter assay, qRT-PCR, Western blot
119	hsa-miR-520g-3p	1	VEGFA	ELISA, Luciferase reporter assay
120	hsa-miR-520h	1	VEGFA	ELISA, Luciferase reporter assay
121	hsa-miR-524-5p	1	MMP9	qRT-PCR, Western blot
122	hsa-miR-543	1	NOS3	Luciferase reporter assay, qRT-PCR
123	hsa-miR-544a	1	STAT3	Luciferase reporter assay, qRT-PCR, Western blot
124	hsa-miR-550a-5p	1	CPEB4	Immunohistochemistry, Luciferase reporter assay, qRT-PCR, Western blot
125	hsa-miR-613	1	BDNF	Luciferase reporter assay, qRT-PCR, Western blot
126	hsa-miR-625-5p	1	HMGA1	Luciferase reporter assay
127	hsa-miR-718	1	VEGFA	Luciferase reporter assay, Western blot
128	hsa-miR-874-3p	1	STAT3	Immunohistochemistry, Luciferase reporter assay, qRT-PCR, Western blot
129	hsa-miR-9-3p	1	MMP9	Western blot
130	hsa-miR-9-5p	1	IL6	Luciferase reporter assay
131	hsa-miR-92a-3p	1	STAT3	Luciferase reporter assay, qRT-PCR, Western blot
132	hsa-miR-96-5p	1	BDNF	Luciferase reporter assay, qRT-PCR
133	hsa-miR-98-5p	1	IL6	Luciferase reporter assay, qRT-PCR, Western blot

Table S2. Description of the signaling pathways and functions of potential targets associated with overweight and obesity.

Potential Target	Target description	Signaling pathway	Function
CCND1	Cyclin D1	Cell Cycle, Wnt/β-catenin, PI3K/AKT and MAPK signaling pathway and Apoptosis	Controls adipocyte proliferation and lipid accumulation.
CCND2	Cyclin D2		Regulates preadipocyte proliferation and adipogenesis.
CCNE1	Cyclin E1		Influences adipocyte proliferation and metabolic function.
CDC25A	Cell division cycle 25A		Promotes adipocyte growth and lipid storage.
CDK6	Cyclin dependent kinase 6		Controls preadipocyte proliferation and obesity-related fat accumulation.
E2F1	E2F transcription factor 1		Influences adipocyte proliferation and metabolic regulation.
E2F2	E2F transcription factor 2		Regulates lipid metabolism and adipogenesis.
IL6	Interleukin 6	Inflammation Pathway and immune response	Chronic low-grade inflammation is a hallmark of obesity. IL6 and STAT3 are key mediators of inflammatory signaling and in conjunction with SOCS6 regulate immune function, while VEGFA contributes to vascular inflammation and remodeling
SOCS6	Suppressor of cytokine signaling 6		
STAT3	Signal transducer and activator of transcription 3		
VEGFA	Vascular endothelial growth factor A		
MAFB	MAF bZIP transcription factor B	Transcription regulation and Wnt/β-catenin, and PI3K/AKT signaling pathway	Modulates lipid metabolism and adipocyte differentiation
MYB	MYB proto-oncogene, transcription factor		Regulates adipogenesis and lipid metabolism
MYC	MYC proto-oncogene, bHLH transcription factor		Modulates adipogenesis and obesity-related metabolic pathways
SOX5	SRY-box 5		Modulates adipocyte differentiation
BCL2	B-cell lymphoma 2	PI3K/AKT signaling pathway and Stress Response	Regulates adipocyte survival, preventing obesity-induced apoptosis.
FOXO3	Forkhead box O3		Modulates adipocyte differentiation and oxidative stress response.
PTEN	Phosphatase and tensin homolog		Regulates adipocyte differentiation and insulin sensitivity.
RECK	Reversion inducing cysteine rich protein with kazal motifs	TGF-β signaling pathway and Transcriptional Regulation	Modulates adipose tissue extracellular matrix composition.
SMAD4	SMAD family member 4		Regulates adipogenesis and lipid metabolism.
TGFBR2	Transforming growth factor beta receptor 2		Inhibits adipogenesis and promotes fibrosis in adipose tissue.
BDNF	Brain derived neurotrophic factor	Neurotrophic Pathways	BDNF is involved in neuronal growth and energy balance regulation.
PAX6	Paired box 6		PAX6 has roles in neural development, potentially affecting appetite control
CHEK1	Checkpoint kinase 1	DNA damage response and cell cycle checkpoint regulation	Maintains adipocyte genomic stability and regulates metabolism
WEE1	WEE1 G2 checkpoint kinase		Modulates adipocyte proliferation and genomic stability
EZH2	Enhancer of zeste 2 polycomb repressive complex 2 subunits	Epigenetic regulation	Controls adipogenesis via chromatin modifications
MECP2	Methyl-CpG binding protein 2		Controls energy balance and metabolic homeostasis

HMGA1	High mobility group AT-hook 1	Insulin Signaling, Adipogenesis and Wnt/β-Catenin Pathway	Insulin signaling is crucial for glucose homeostasis. Dysregulation is a key feature of obesity and related metabolic disorders
HMGA2	High mobility group AT-hook 2		HMGA2 negatively regulates the Wnt/β-catenin signaling pathway, promoting adipocyte formation and lipid accumulation.
IGF1R	Insulin like growth factor 1 receptor	MAPK and PI3K/AKT signaling pathway	Modulates insulin signaling and energy metabolism.
KRAS	KRAS proto-oncogene, GTPase		Influences adipocyte proliferation and metabolic signaling.
BMI1	B lymphoma Mo-MLV insertion region 1 homolog	Wnt and NF-κB signaling pathway	Controls adipocyte stem cell renewal and differentiation.
CPEB4	Cytoplasmic polyadenylation element binding protein 4	mRNA Translation Control	Regulates specific mRNAs via cytoplasmic polyadenylation, enhancing or repressing their translation.
		Stress and Inflammatory Pathways	Influences stress granule dynamics and inflammation, indirectly contributing to chronic inflammatory states in obesity.
		Adipocyte Differentiation	Involved in pathways regulating the differentiation of pre-adipocytes to adipocytes.
CADM1	Cell adhesion molecule 1	Hippo signaling pathway and Cell Adhesion	Influences adipocyte differentiation and insulin sensitivity.
CHUK	Conserved helix-loop-helix ubiquitous kinase	NF-κB signaling pathway	Modulates inflammation and metabolic regulation in obesity.
DICER1	Ribonuclease III	miRNA Biogenesis	Controls adipogenesis and adipose tissue homeostasis via miRNA regulation.
FOXO1	Forkhead box O1	Insulin and Wnt signaling pathway	Regulates adipogenesis, insulin sensitivity, and lipid metabolism.
JAG1	Jagged 1	Notch signaling pathway, and cell fate determination	Regulates adipocyte differentiation and obesity-related pathways.
VEGFA	Vascular endothelial growth factor A	Angiogenesis	VEGFA is a primary regulator of blood vessel formation, critical in adipose tissue expansion during obesity
YAP1	Yes associated protein 1	Hippo and Wnt/β-Catenin signaling pathway	Regulates adipogenesis and adipose tissue homeostasis.

JAK/STAT: Janus kinase/signal transducer and activator of transcription; MAPK: Mitogen-activated protein kinases; NF-κB: Nuclear Factor kappa B; PI3K/AKT: Apoptosis and Phosphatidylinositol 3-kinase (PI3K)/Protein Kinase B (Akt)
